# Medical Costs and Productivity Losses of Atrial Fibrillation Among US Privately Insured Employees

**DOI:** 10.1001/jamanetworkopen.2025.59227

**Published:** 2026-02-12

**Authors:** Han Zhang, Jun Soo Lee, Sein Kim, Ashutosh Kumar, Yu Wang, Omoye Imoisili, Feijun Luo, Utibe R. Essien

**Affiliations:** 1 Division for Heart Disease and Stroke Prevention, Centers for Disease Control and Prevention, Atlanta, Georgia; 2 Department of Global Health and Population, Harvard T.H. Chan School of Public Health, Boston, Massachusetts; 3 Department of Population Health Sciences, Georgia State University School of Public Health, Atlanta; 4 Division of General Internal Medicine and Health Services Research, David Geffen School of Medicine at the University of California, Los Angeles; 5 Center for the Study of Healthcare Innovation, Implementation & Policy, Greater Los Angeles VA Healthcare System, Los Angeles, California

## Abstract

**Importance:**

Atrial fibrillation (AF) is the most common sustained cardiac arrhythmia and a major factor underlying US health care costs. While its clinical burden is well documented, the full economic impact of AF—particularly among working-age adults with productivity losses—remains underexplored.

**Objective:**

To estimate medical costs and productivity losses associated with AF among privately insured US employees and assess whether this burden varies by sex and rurality.

**Design, Setting, and Participants:**

This cross-sectional study used 2021 Merative MarketScan Commercial Claims and Health and Productivity Management Databases. The sample included adults aged 18 to 64 years with continuous enrollment in noncapitated, employer-sponsored insurance and no pregnancy-related diagnoses. Analyses were conducted during January to July 2025.

**Exposures:**

Diagnosis of AF, defined by 1 or more inpatient or emergency department claims or 2 or more outpatient claims with *International Classification of Disease, Tenth Revision, Clinical Modification* code I48.

**Main Outcomes and Measures:**

Primary outcomes were total all-cause annual medical costs and productivity losses. Medical costs were disaggregated into emergency department, inpatient, outpatient, and prescription costs. Productivity losses included sick leave, short-term disability, and long-term disability; days were observed directly, and dollar-valued costs were estimated by applying national average wages. All outcomes were prespecified. Propensity score overlap weighting was applied to balance covariates.

**Results:**

Among 1 612 398 individuals (mean [SD] age, 44.00 [11.11] years; 623 335 female [38.66%]; 1 489 709 [92.39%] living in an urban area), 10 190 (0.63%) were diagnosed with AF. AF was associated with $11 392.55 (95% CI, $10 649.70-$12 135.38) in incremental annual medical costs, primarily from outpatient care ($7058.81 [95% CI, $6563.89-$7553.72] for services and $1874.58 [95% CI, $1600.61-$2148.56] for prescriptions). Compared with those without AF, those with AF had 0.97 (95% CI, 0.02-1.93) excess sick leave days and 2.93 (95% CI, 2.14-3.72) excess short-term disability days, translating to productivity-related costs of $269.81 (95% CI, $144.65-$397.77) for sick leave and $570.51 (95% CI, $471.21-$669.81) for short-term disability. Long-term disability outcomes did not differ significantly. Females incurred higher AF-related emergency (mean difference, $422.61; 95% CI, $178.32-$666.89) and inpatient care costs (mean difference, $1588.67; 95% CI, $466.23-$2711.12) than males.

**Conclusions and Relevance:**

In this cross-sectional study of privately insured employees, AF was associated with $11 393 in higher medical costs per person, with outpatient care accounting for the largest share, and $840 in higher productivity losses per person. These findings underscore the need to improve outpatient treatment and reduce AF-related workplace disruptions for working-age adults.

## Key Points

**Question:**

What are the medical costs and productivity losses associated with atrial fibrillation (AF) among privately insured US employees?

**Findings:**

In this cross-sectional study of 1 612 398 US adults aged 18 to 64 years with employer-sponsored insurance, persons with AF incurred $11 392.55 more in excess annual medical costs—primarily from outpatient care ($7058.81 for services and $1874.58 for prescriptions)—and $840.32 more in excess productivity-related costs from sick leave and short-term disability than those without AF.

**Meaning:**

This study found that AF was associated with a substantial economic burden among working-age adults through increased outpatient care costs and workplace productivity losses, suggesting opportunities for targeted interventions in outpatient treatment and workplace support.

## Teaser

This cross-sectional study estimates medical costs and productivity losses associated with atrial fibrillation (AF) among privately insured employees in the US.

## Introduction

Atrial fibrillation (AF) is the most common sustained cardiac arrhythmia,^1,2^ affecting more than 10 million US adults, and is projected to rise through 2050.^3,4^ AF elevates the risk of ischemic stroke, heart failure, and myocardial infarction, contributing to substantial clinical and economic burden.^5^ People with AF have a 5-fold higher risk of ischemic stroke,^6^ which alone accounted for $56.2 billion in US health care spending in 2019 to 2020.^7^

As AF care evolves, understanding the economic burden of AF and differences across populations can guide health care planning and payer decision-making. Prior US studies, mostly using pre-2015 data, estimated annual AF-related medical costs at $3632 to $27 896 per patient.^8,9,10^ These estimates predate major advances in AF care, including direct oral anticoagulants,^11^ early rhythm-control therapy,^12^ increased catheter ablation use,^13^ and expanded outpatient monitoring.^14^ AF burden also varies across populations; women experienced more severe symptoms, diagnostic delays, and distinct treatment trajectories,^15,16,17,18^ and rural residents face barriers to AF awareness, diagnosis, and timely outpatient care.^19,20^ These patterns underscore the need for updated, disaggregated AF cost estimates to guide targeted interventions and resource allocation.

Although more prevalent among older adults,^7,21^ AF also affects working-age individuals through productivity losses from absenteeism, disability, and early workforce exit.^22,23^ These disruptions may lead to financial hardship for patients and economic strain on employers, insurers, and public programs.^24^ Contemporary US estimates of productivity losses are limited.^15^ Existing studies, mostly before 2010, suggest productivity losses represent 13% of AF-related costs in the US,^24,25^ while international estimates range widely from less than 5% (Mexico,^26^ Spain,^27^ and Denmark^28^) to approximately 20% (Portugal,^29^ the Netherlands,^30^ and Sweden^31^), to greater than 40% (Germany^32^). These differences reflect measurement challenges and cross-country variations in labor markets, social insurance, and health systems. Meanwhile, workforce shifts—rising participation among older adults, growing reliance on employer-sponsored benefits, and geographic variations in job structures^33^—highlight the need for updated, US-specific estimates. Because many US employers offer health insurance, paid sick leave, and disability benefits,^34^ a clearer understanding of productivity losses due to AF may be useful in informing workplace and public health policy.

Given rising AF prevalence^3,4^ and changes in treatment strategies^12,14^ and workforce composition,^33^ this study quantifies the current economic burden of AF among working-age US adults with employer-sponsored insurance. Specifically, we estimated the medical costs and productivity losses associated with AF by comparing individuals with and without AF who were similar in demographics and comorbidities. We also examined differences by sex and rurality to inform targeted strategies to reduce the economic burden of AF.

## Methods

### Data Source

This cross-sectional study used 2021 Merative MarketScan Commercial Claims and Encounters Data linked to the Health and Productivity Management (HPM) Database. The Commercial Database includes enrollment records, medical and pharmacy claims, and costs for employees and dependents with employer-sponsored insurance across more than 300 employers, 30 health plans, and 500 hospitals.^35^ The HPM subset includes payroll-based absenteeism and disability records.^35^ This secondary analysis of deidentified data involved no participant interaction and, in accordance with the Common Rule (45 CFR 46.102), did not require institutional review board approval or informed consent. Reporting follows the Strengthening the Reporting of Observational Studies in Epidemiology (STROBE) reporting guideline for cross-sectional studies.

### Sample Selection

Adults aged 18 to 64 years with continuous enrollment in 2021 were included. Individuals with pregnancy-related diagnoses (codes in eTable 1 in Supplement 1) were excluded due to distinct, high-intensity care patterns that could obscure differences in costs between individuals with and without AF. We also excluded individuals in capitated plans where payments are fixed per enrollee rather than based on service use. To estimate productivity losses, we restricted the sample to those with HPM absence data; missing HPM reflects structural employer nonreporting rather than no leaves or disability. We constructed outcome-specific analysis sets, each including only individuals with complete data for that outcome: medical costs, sick leave, short-term disability, and long-term disability. As a result, the analysis sets are not identical across outcomes.

### Measures

The exposure variable was a diagnosis of AF, identified by 1 or more inpatient or emergency department claims or 2 or more outpatient claims with *International Classification of Disease, Tenth Revision, Clinical Modification* (*ICD-10-CM*) code I48 in 2021.

Outcome variables included (1) total medical costs, defined as the total annual insurer- and patient-paid amounts during 2021, disaggregated into emergency department (ED) care, inpatient care (including inpatient medications), outpatient services, and pharmacy claims for outpatient prescriptions only (laboratory test costs are not separately listed due to its minor share); (2) healthcare utilization, measured as counts of ED visits, inpatient admissions, outpatient visits, and prescription fills; and (3) productivity losses, assessed by days lost and monetary value. Days reflected workdays missed due to sick leave, short-term disability, and long-term disability in employer-reported absence records. In rare cases where reported days exceeded 365 within a year (eg, due to overlapping claims or reporting errors), values were capped at 365 per category to reflect annual maximums. Dollar-valued productivity losses equaled days × 8 hours per day × the 2021 US average hourly wage ($30.61) for private nonfarm employees (seasonally adjusted) from the US Bureau of Labor Statistics.^36^ To account for partial wage replacement typically paid during disability periods, adjustment factors of 70% for short-term disability and 60% for long-term disability were applied, consistent with prior literature.^37,38^ All outcomes were all-cause and reported as adjusted incremental differences between adults with and without AF. Following published recommendations,^39^ costs are converted to 2024 US dollars using the Personal Consumption Expenditures Health Price Index for medical costs^40^ and the Employment Cost Index for productivity-related costs.^41^

Covariates included demographic characteristics (age group, sex, rurality, and region), clinical risk factors and comorbidities (alcohol use, tobacco use, obesity, hypertension, hyperlipidemia, COVID-19 infection, myocardial infarction, congestive heart failure, peripheral vascular disease, chronic pulmonary disease, rheumatic disease, peptic ulcer disease, diabetes, hemiplegia or paraplegia, kidney disease, any malignant tumor, liver disease, and HIV or AIDS). Rurality was classified using county-level metropolitan vs nonmetropolitan designations,^35,42^ and region was classified as Northeast, Midwest, South, and West. Risk factors and comorbidities were identified using *ICD-10-CM* codes (eTable 2 in Supplement 1) and selected based on established AF risk factors from clinical guidelines^11^ and validated Charlson Comorbidity Index components^43^ and reviewed by clinical experts to ensure relevance. We screened for multicollinearity using pairwise correlations (ρ) among comorbidities and risk factors (threshold, |ρ|>0.80); none exceeded this threshold (eTable 3 in Supplement 1).

### Statistical Analysis

#### Primary Analysis

We summarized baseline characteristics and unadjusted outcomes by AF status using Wilcoxon rank-sum tests (continuous) and Pearson χ^2^ (categorical) tests. To improve comparability between individuals with and without AF, we applied propensity score overlap weighting (OW), which targets the population with covariate overlap. Propensity scores were estimated using logistic regression with iterative maximum likelihood, incorporating higher-order interactions of baseline covariates (eTable 4 and eTable 5 in Supplement 1).^44,45^ Individuals without AF were weighted by their estimated propensity score, and individuals with AF were weighted by 1 minus the propensity score.^46,47,48,49^ We used normalized OW weights so the weighted sample size equaled the original sample size. OW was selected over inverse probability weighting and weighting schemes targeting the average treatment effect on the treated because it estimates the average treatment effect in the overlap population and yields bounded, stable weights with strong covariate balance and minimal extrapolation, consistent with best practices in the causal inference literature.^46^ Comparative performance across methods is shown in eTable 6, eTable 7, and eFigures 1 to 4 in Supplement 1.

Medical costs were modeled using 2-part models to account for right-skewed distributions and the presence of zero costs in some categories (eg, inpatient or ED). The first part used logistic regression to estimate the probability of incurring any cost, and the second part used a generalized linear model with a log-link and gamma distribution to estimate cost levels among individuals with nonzero values.^50^ Count-based outcomes (utilization and productivity loss days) used zero-inflated negative binomial models to accommodate overdispersion and excess zeros.^51^ All models adjusted for the full covariates set, with results reported as average marginal effects by AF status and the adjusted differences per-person. Subgroup analyses by sex and rurality (prespecified due to well-documented disparities in AF care and access) used the same OW and outcome models; covariate balance remained satisfactory within subgroups. Between-group differences were tested using AF × subgroup interactions with Wald tests. All tests were 2-sided with α = .05. Analyses were conducted from January to July 2025 and used Stata MP version 18 (StataCorp).

#### Sensitivity Analysis

Robustness of modeling strategy was assessed in 2 ways. First, we reestimated medical costs using generalized linear model (log-link and gamma distribution) and count outcomes using standard negative binomial regressions, and second, we applied alternative weighting approaches (unweighted, inverse probability weighting, and average treatment effect on the treated).

## Results

### Study Population and Descriptive Statistics

**Figure 1.**
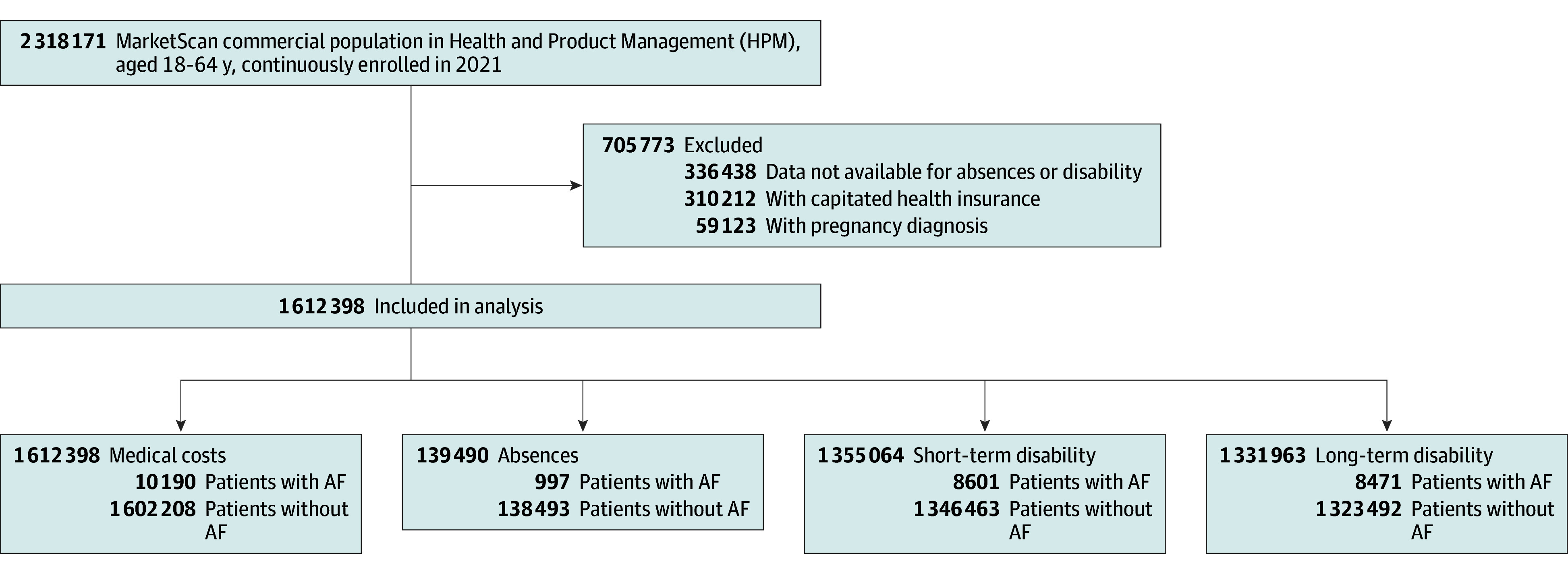
Study Sample Selection of Patients Diagnosed With Atrial Fibrillation (AF), MarketScan Commercial and Health and Product Management Database, 2021

The analytic sample for medical costs included 1 612 398 working-age adults (mean [SD] age, 44.00 [11.11] years; 623 335 female [38.66%]; 1 489 709 [92.39%] living in an urban area); the sample included 139 490 adults for sick leave, 1 355 064 adults for short-term disability, and 1 331 963 adults for long-term disability (Figure 1). Of the 1 612 398 adults, 10 190 (0.63%) had a diagnosis of AF and 1 602 208 (99.37%) did not. Compared with those without AF, people with AF were older (mean [SD] age, 54.28 [7.65] vs 43.93 [11.09] years; *P* < .001), less likely to be female (2051 individuals [20.13%] vs 621 284 individuals [38.78%]; *P* < .001), and had a higher prevalence of cardiovascular and metabolic conditions, including hypertension (6147 individuals [60.32%] vs 209 333 individuals [13.07%]; *P* < .001), diabetes (2025 individuals [19.87%] vs 93 734 individuals [5.85%]; *P* < .001), myocardial infarction (659 individuals [6.47%] vs 7584 individuals [0.47%]; *P* < .001), and obesity (2745 individuals [26.94%] vs 106 705 individuals [6.66%]; *P* < .001) (Table 1).

**Table 1.** Sample Characteristics, Health Care Cost, Utilization, and Productivity Loss, Overall and by AF Diagnosis Status, MarketScan Commercial and Health and Product Management Database, 2021^a^

Characteristic	Participants, No. (%)			*P* value
	All (N = 1 612 398)	Without AF (n = 1 602 208)	With AF (n = 10 190)	
Demographic characteristics				
Age, mean (SD), y	44.00 (11.11)	43.93 (11.09)	54.28 (7.65)	<.001
Age group, y				
18-34	394 617 (24.47)	394 339 (24.61)	278 (2.73)	<.001
35-44	411 201 (25.50)	410 296 (25.61)	905 (8.88)	<.001
45-54	451 159 (27.98)	448 242 (27.98)	2917 (28.63)	.15
55-64	355 421 (22.04)	349 331 (21.80)	6090 (59.76)	<.001
Sex				
Female	623 335 (38.66)	621 284 (38.78)	2051 (20.13)	<.001
Male	989 063 (61.34)	980 924 (61.22)	8139 (79.87)	<.001
Urban residency	1 489 709 (92.39)	1 480 491 (92.40)	9218 (90.46)	<.001
Census region				
Northeast	239 802 (14.87)	238 197 (14.87)	1605 (15.75)	.01
Midwest	387 301 (24.02)	384 474 (24.00)	2827 (27.74)	<.001
South	694 461 (43.07)	690 136 (43.07)	4325 (42.44)	.20
West	283 790 (17.60)	282 384 (17.62)	1406 (13.80)	<.001
Risk factors and comorbidities				
Alcohol use	8758 (0.54)	8490 (0.53)	268 (2.63)	<.001
Tobacco use	22 089 (1.37)	21 555 (1.35)	534 (5.24)	<.001
Obesity	109 450 (6.79)	106 705 (6.66)	2745 (26.94)	<.001
Hypertension	215 480 (13.36)	209 333 (13.07)	6147 (60.32)	<.001
Hyperlipidemia	169 986 (10.54)	165 810 (10.35)	4176 (40.98)	<.001
Myocardial infarction	8243 (0.51)	7584 (0.47)	659 (6.47)	<.001
Congestive heart failure	8648 (0.54)	6809 (0.42)	1839 (18.05)	<.001
Peripheral vascular	7538 (0.47)	6563 (0.41)	975 (9.57)	<.001
Chronic pulmonary disease	36 105 (2.24)	35 258 (2.20)	847 (8.31)	<.001
Rheumatic disease	9912 (0.61)	9778 (0.61)	134 (1.32)	<.001
Peptic ulcer disease	1322 (0.08)	1276 (0.08)	46 (0.45)	<.001
Diabetes	95 759 (5.94)	93 734 (5.85)	2025 (19.87)	<.001
Hemiplegia or paraplegia	1284 (0.08)	1182 (0.07)	102 (1.00)	<.001
Kidney disease	10 807 (0.67)	10 290 (0.64)	517 (5.07)	<.001
Malignant tumor	25 244 (1.57)	24 721 (1.54)	523 (5.13)	<.001
Liver disease	14 169 (0.88)	13 804 (0.86)	365 (3.58)	<.001
HIV or AIDS	3447 (0.21)	3421 (0.21)	26 (0.26)	.36
COVID-19 infection	144 896 (8.99)	143 489 (8.96)	1407 (13.81)	<.001
Medical cost and productivity loss, mean (SD)^b^				
Total medical costs, $^c^	8334.63 (30 651.70)	8045.60 (28 956.24)	53 780.35 (118 667.31)	<.001
ED costs, $	531.71 (2617.13)	521.69 (2558.94)	2106.87 (7191.24)	<.001
Inpatient costs, $	1410.73 (17 957.45)	1272.12 (15 993.15)	23 202.82 (101 642.99)	<.001
Outpatient service costs, $	3870.60 (16 036.53)	3766.20 (15 668.24)	20 287.47 (42 685.45)	<.001
Pharmacy costs, $	2184.37 (12 541.77)	2151.19 (12 450.66)	7401.03 (22 089.83)	<.001
No. of ED visits	0.21 (0.71)	0.20 (0.70)	0.69 (1.66)	<.001
No. of inpatient admissions	0.03 (0.24)	0.03 (0.23)	0.40 (0.81)	<.001
No. of outpatient visits	9.29 (12.95)	9.20 (12.83)	23.41 (21.91)	<.001
No. of pharmacy prescriptions	11.53 (15.00)	11.40 (14.83)	32.16 (24.16)	<.001
No. of sick leave, d	2.40 (9.42)	2.37 (9.28)	6.07 (20.95)	<.001
No. of short-term disability, d	3.14 (18.50)	3.08 (18.27)	12.61 (40.21)	<.001
No. of long-term disability, d	0.46 (11.04)	0.44 (10.83)	2.89 (28.97)	<.001
Sick leave payment, $	666.57 (2620.94)	659.21 (2582.04)	1688.77 (5828.24)	<.001
Short-term disability payment, $	611.38 (3602.67)	599.60 (3556.50)	2455.30 (7830.13)	<.001
Long-term disability payment, $	76.22 (1842.99)	73.62 (1807.68)	481.91 (4834.82)	<.001

Abbreviations: AF, atrial fibrillation; ED, emergency department.

^a^ Wilcoxon nonparametric rank-sum test was used to test differences in means for continuous variables, and the Pearson χ^2^ was used to test differences in proportions for categorical variables by AF diagnosis status.

^b^ Medical costs and productivity costs were adjusted to 2024 USD using the Personal Consumption Expenditures Health Price Index from the US Bureau of Economic Analysis,^40^ and the Employment Cost Index (Total Compensation for Civilian Workers) from the US Bureau of Labor Statistics,^41^ respectively.

^c^ Laboratory test costs are included within total medical costs and not separately reported due to their small amounts.

Before adjustment, individuals with AF had markedly higher annual medical costs, including mean (SD) total ($53 780.35 [$118 667.31] vs $8045.60 [$28 956.24]; *P* < .001), ED ($2106.87 [$7191.24] vs $521.69 [$2558.94]; *P* < .001), inpatient ($23 202.82 [$101 642.99] vs $1272.12 [$15 993.15]; *P* < .001), outpatient ($20 287.47 [$42 685.45] vs $3766.20 [$15 668.24]; *P* < .001), and pharmacy ($7401.03 [$22 089.83] vs $2151.19 [$12 450.66]; *P* < .001). Overall, the mean difference in medical costs between those with and without AF was $45 734.75 (95% CI, $45 141.92-$46 327.59). Individuals with AF also had a higher mean (SD) number of sick leave days (6.07 [20.95] vs 2.37 [9.28] days; *P* < .001), short-term disability (12.61 [40.21] vs 3.08 [18.27] days; *P* < .001), and long-term disability (2.89 [28.97] vs 0.44 [10.83] days; *P* < .001).

### Adjusted Economic Burden of AF

After adjustment (Table 2), AF was associated with $11 392.55 (95% CI, $10 649.70-$12 135.38) higher annual medical costs. Outpatient services accounted for the largest share (mean difference, $7058.81; 95% CI, $6563.89-$7553.72), followed by inpatient care (mean difference, $3626.52; 95% CI, $3097.28-$4155.74), pharmacy (mean difference, $1874.58; 95% CI, $1600.61-$2148.56), and ED costs (mean difference, $889.80; 95% CI, $778.82-$1000.79). Adjusted annual medical cost averaged $19 545.48 (95% CI, $18 632.26-$20 458.70) for individuals with AF, compared with $8152.93 (95% CI, $7883.29-$8422.58) for those without AF. Adjustment substantially narrowed the cost gap from unadjusted comparisons ($53 780.35 for AF and $8045.60 for non-AF). AF was also associated with greater health care utilization, including 0.29 (95% CI, 0.26-0.32) excess ED visits , 0.16 (95% CI, 0.15-0.18) excess inpatient admissions, 7.40 (95% CI, 7.01-7.79) excess outpatient visits, and 8.50 (95% CI, 8.06-8.95) excess prescriptions filled annually.

**Table 2.** Adjusted Medical Cost and Productivity Loss Associated With Atrial Fibrillation, MarketScan Commercial and Health and Product Management Database, 2021^a^

Category	Patients, No.	Adjusted mean (95% CI)		Adjusted mean difference (95% CI)	*P* value
		Without AF	With AF		
Medical costs^b^					
Total medical costs, $	1 612 398	8152.93 (7883.29 to 8422.58)	19 545.48 (18 632.26 to 20 458.70)	11 392.55 (10 649.70 to 12 135.38)	<.001
ED costs, $	1 612 398	859.36 (787.08 to 931.62)	1749.16 (1599.14 to 1899.17)	889.80 (778.82 to 1000.79)	<.001
Inpatient costs, $	1 612 398	2151.85 (1958.32 to 2345.37)	5778.35 (5119.79 to 6436.92)	3626.52 (3097.28 to 4155.74)	<.001
Outpatient service costs, $	1 612 398	4543.23 (4399.83 to 4686.62)	11 602.04 (11 026.77 to 12 177.29)	7058.81 (6563.89 to 7553.72)	<.001
Pharmacy costs, $	1 612 398	2247.35 (2165.95 to 2328.76)	4121.93 (3802.55 to 4441.31)	1874.58 (1600.61 to 2148.56)	<.001
No. of ED visits	1 612 398	0.39 (0.39 to 0.40)	0.68 (0.66 to 0.71)	0.29 (0.26 to 0.32)	<.001
No. of inpatient admissions	1 612 398	0.20 (0.20 to 0.21)	0.37 (0.36 to 0.38)	0.16 (0.15 to 0.18)	<.001
No. of outpatient visits	1 612 398	16.49 (16.37 to 16.61)	23.89 (23.49 to 24.28)	7.40 (7.01 to 7.79)	<.001
No. of pharmacy prescriptions	1 612 398	24.09 (23.96 to 24.22)	32.59 (32.16 to 33.02)	8.50 (8.06 to 8.95)	<.001
Productivity loss^c^					
No. of sick leave d	139 490	4.82 (4.22 to 5.41)	5.79 (4.74 to 6.84)	0.97 (0.02 to 1.93)	.05
No. of short-term disability d	1 355 064	8.98 (8.73 to 9.23)	11.91 (11.15 to 12.67)	2.93 (2.14 to 3.72)	<.001
No. of long-term disability d	1 331 963	2.17 (1.94 to 2.39)	2.57 (2.01 to 3.14)	0.41 (–0.20 to 1.02)	.19
Sick leave payment, $	139 490	1340.73 (1173.83 to 1504.84)	1610.55 (1318.48 to 1902.61)	269.81 (144.65 to 397.77)	.05
Short-term disability payment, $	1 355 064	1748.52 (1699.83 to 1797.19)	2319.01 (2171.04 to 2467.00)	570.51 (471.21 to 669.81)	<.001
Long-term disability payment, $	1 331 963	361.92 (335.94 to 369.57)	427.10 (334.78 to 696.46)	68.43 (–33.38 to 170.24)	.19

Abbreviations: AF, atrial fibrillation; ED, emergency department.

^a^ Outcomes are all-cause and reported per person-year. AF and non-AF groups were balanced using propensity-score overlap weighting. Count outcomes (health care utilization, days of sick leave, short-term disability, and long-term disability) were modeled with zero-inflated negative binomial regression to address overdispersion and excess zeros. Models were adjusted for age, sex, urbanicity, census region, risk factors, and comorbidities. The Wilcoxon nonparametric rank-sum test was used to test the differences in means for continuous variables by AF diagnosis status.

^b^ Medical costs were estimated with 2-part models and were converted to 2024 USD using the Personal Consumption Expenditures Health Price Index from the US Bureau of Economic Analysis. Because total and component costs were estimated separately with nonlinear links, and laboratory tests were not reported as a standalone category, components may not sum exactly to totals.

^c^ Dollar values for productivity losses were calculated as: days × 8 hours × the 2021 average hourly wage for private nonfarm employees (US Bureau of Labor Statistics),^36^ valuing sick leave at 100% and applying wage-replacement factors of 70% (short-term disability) and 60% (long-term disability). Values were converted to 2024 USD using the Employment Cost Index (Total Compensation for Civilian Workers) from the US Bureau of Labor Statistics.^41^

For productivity losses, individuals with AF had 0.97 (95% CI, 0.02-1.93) more sick leave days and 2.93 (95% CI, 2.14-3.72) more short-term disability days annually compared with those without AF, equivalent to $269.81 (95% CI, $144.65-$397.77) in sick leave and $570.51 (95% CI, $471.21-$669.81) in short-term disability. Differences in long-term disability were not statistically significant. Sensitivity analyses using alternative models and weighting approaches yielded consistent results (eTables 8-11 in Supplement 1). Overall, AF was associated with $11 393 in excess annual medical costs per person and $840 in excess productivity losses per person.

### Subgroup Analyses by Sex and Rurality

**Figure 2.**
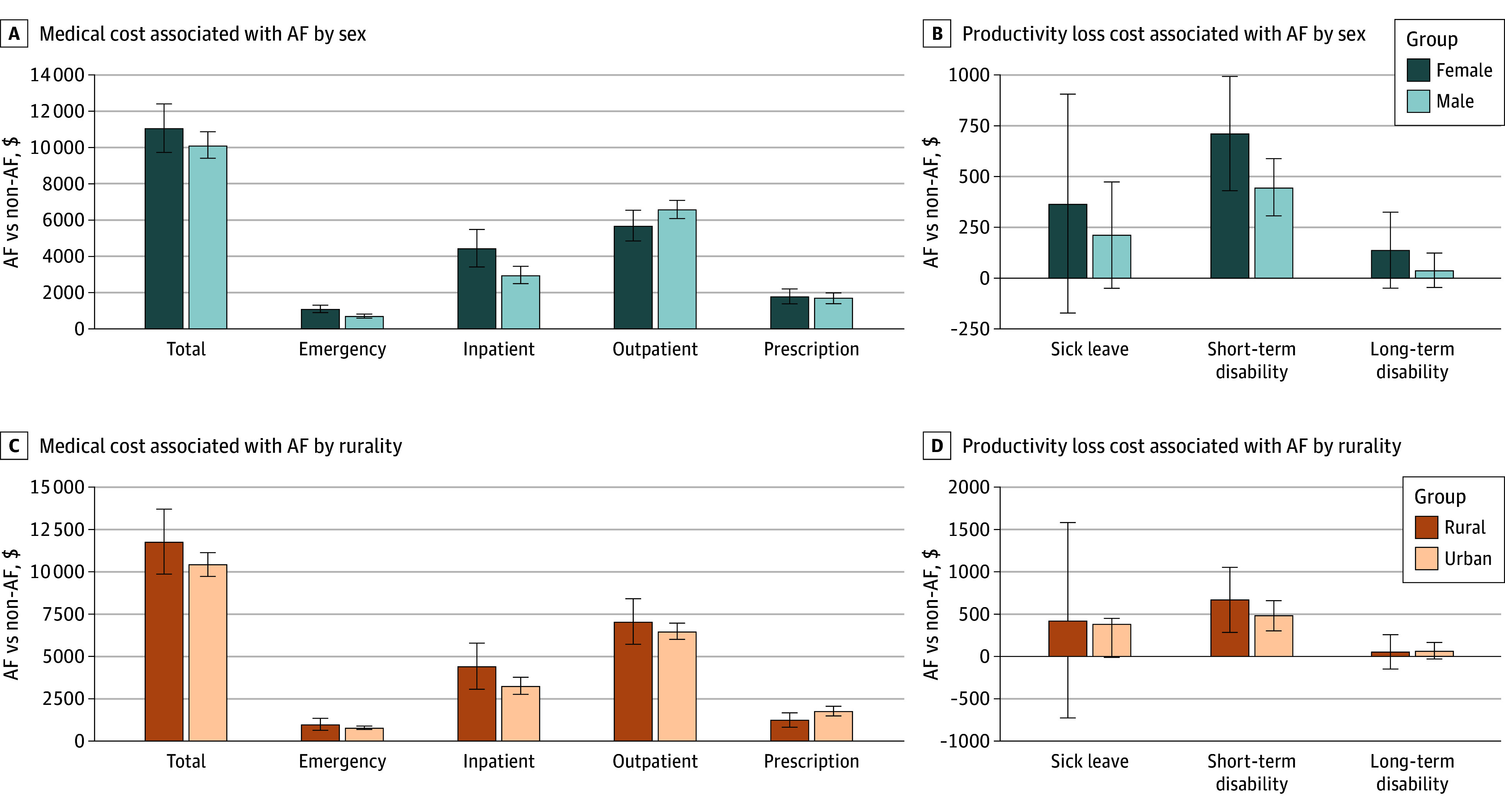
Adjusted Differences in Medical Costs and Productivity Losses Among Individuals With Atrial Fibrillation (AF), Stratified by Sex and Rurality, MarketScan Commercial and Health and Product Management Database, 2021 This figure presents the adjusted economic burden associated with AF by sex (male and female) and place of residence (urban and rural). A, Incremental total annual medical costs. B, Differences in productivity-related costs due to sick leave, short-term disability, and long-term disability. C, Incremental medical cost by category, including emergency department visits, inpatient admissions, outpatient visits, and prescription fills. D, Differences in the number of days lost due to sick leave, short-term disability, and long-term disability. All estimates reflect adjusted mean differences between individuals with and without AF, with 95% CIs indicated by error bars.

Figure 2 and eTables 12 and 13 in Supplement 1 show adjusted AF-related economic burden by sex and rurality. AF was associated with substantial excess medical costs and productivity losses for both male and female sex, although the cost distribution differed. Among males, AF was associated with a mean of $10 933.50 (95% CI, $10 149.84 to $11 717.17) in higher annual medical costs, ranging from $765.07 (95% CI, $653.08 to $877.05) in ED to $7101.16 (95% CI, $6556.01 to $7646.31) in outpatient services. Among females, AF was associated with $11 930.83 (95% CI, $10 486.54 to $13 375.13) in excess medical costs, ranging from $1187.68 (95% CI, $956.57 to $1418.79) in ED to $6141.00 ($5244.53 to $7037.47) in outpatient services. Compared with males, females incurred higher ED (mean difference, $422.61; 95% CI, $178.32 to $666.89) and inpatient (mean difference, $1588.67; 95% CI, $466.23 to $2711.12) costs, due to more ED visits (mean difference, 0.14 visits; 95% CI, 0.07 to 0.22 visits) and inpatient admissions (mean difference, 0.04 admissions; 95% CI, 0.01 to 0.08 admissions). Females also had more outpatient visits (mean difference, 1.35 visits; 95% CI, 0.29 to 2.42 visits), yet outpatient service costs were not statistically different ($–960.16; 95% CI, –$1966.23 to $45.91). Productivity losses from short-term disability among females were $811.94 (95% CI, $490.68 to $1133.22) vs $510.15 (95% CI, $350.48 to $671.76) for males, with a nonsignificant difference of $301.80 (95% CI, –$27.51 to $630.77).

Urban and rural residents with AF had cost increases vs their counterparts without AF, with minimal rural-urban differences. Urban residents with AF had $11 281.09 (95% CI, $10 509.59-$12 052.60) higher annual medical costs, with differences ranging from $871.44 (95% CI, $759.11-983.75) in ED to $7011.93 (95% CI, $6495.76-$7528.10) in outpatient services compared with urban residents without AF. Rural residents with AF had $12 735.93 (95% CI, $10 670.29-$14 801.56) higher annual medical costs, with differences ranging from $1081.87 (95% CI, $683.90-$1479.85) in ED to $7659.64 (95% CI, $6216.54-$9102.75) in outpatient services compared with rural residents without AF. The only significant rural-urban difference was $556.47 (95% CI, $25.43–$1087.53) higher pharmacy costs in urban residents. AF-related productivity losses from short-term disability for urban patients ($547.14; 95% CI, $389.42-$706.80) and rural patients ($761.33; 95% CI, $325.16-$1199.42) were not statistically different.

## Discussion

This cross-sectional study offers an updated and comprehensive assessment of the economic burden of AF among privately insured US employees aged 18 to 64 years. Using linked national claims and payroll-based absence records, we estimated both medical costs and productivity losses associated with AF in employment settings. After adjustment for demographic and clinical characteristics, AF was associated with an excess economic burden of $12 234 per-person annually—$11 393 in excess medical costs and $840 in productivity losses.

Our per-person incremental costs were lower than American Heart Association national estimates,^7^ which are unadjusted averages reflecting older populations, broader payer mixes, and more inpatient use. Prior cost-burden studies likewise reported higher costs in older or Medicare-enrolled groups,^8,10,15,52,53^ potentially reflecting differences in study population and analytical approach. For example, Deshmukh et al^10^ reported AF-related costs exceeding $27 000 annually in an older commercially insured cohort (mean age, 73 years), whereas our population was younger and had access to employer-sponsored benefits. We also adjusted for a more granular set of comorbidities (rather than composite indexes),^10,25,27,54^ narrowing the unadjusted cost gap from $45 735 to $11 393 and enabling comparisons among more clinically similar patients. Building on prior propensity-score matching approaches,^8,10^ we used OW with models tailored for skewed cost data, which preserved all observations, reduced influences from extreme values, and improved covariate balance.^50,55^ Together, these strategies likely produced conservative, lower-bound estimates of the economic burden of AF in this employed privately insured working-age population.

A notable difference from earlier work is cost composition; outpatient care (including services and prescriptions) now accounts for most AF-related spending, contrasting with prior inpatient-dominant profiles.^8,10,15,56^ This finding may reflect evolving care patterns (eg, increased use of oral anticoagulants,^57^ outpatient rhythm-management procedures such as catheter ablation,^12,13^ and remote monitoring^11^) and the younger age profile of our cohort, for whom AF hospitalizations are less frequent and diagnostic evaluation and rhythm-control therapies are typically delivered in ambulatory settings.^58^ These patterns suggest that outpatient benefit design, pharmacy coverage, and access to specialty outpatient care may warrant prioritization for chronic AF management.

Payroll-tracked absenteeism allowed us to quantify productivity losses—an often-underrepresented dimension of AF burden. Although smaller than medical costs, productivity losses averaged $840 per person annually; across large workforces, even modest per-person differences can accrue to meaningful organizational impacts. These losses underscore the significant workplace impact of AF and the importance of engaging employers in prevention, timely diagnosis, and workplace accommodations.^59,60,61^

Subgroup analyses revealed notable sex-based differences but little rural-urban variation. Compared with males, females with AF had higher ED and inpatient costs and greater utilization, despite more outpatient visits but similar outpatient service spending. This pattern aligns with evidence that females with AF—especially with atypical or no symptoms—received less intensive treatments (eg, rhythm control or cardioversion),^10,16,17,62^ potentially contributing to more acute episodes and downstream costs. Rural residents had lower pharmacy costs, possibly reflecting differences in access, prescribing, or affordability.^63,64^ Other cost categories were similar to urban residents, possibly because limited local access directs complex cases to urban facilities where costs converge.^65,66^ Additionally, because claims only capture care seekers, rural patients in our dataset may represent a sicker subset, attenuating urban-rural differences.^67,68^ Productivity losses did not differ by sex or rurality, potentially reflecting standardized leave and disability benefits in this insured workforce.

Overall, AF imposes substantial economic burdens on patients, employers, and the health care system, primarily through outpatient care and partly through productivity losses. For health systems and payers, high outpatient costs may inform strategies to improve ambulatory access and chronic disease management via integrated care that combines clinic visits, rhythm monitoring, and coordinated follow-up.^69^ For employers, productivity losses highlight the potential benefits of workplace wellness initiatives, health screenings, and accommodations to mitigate AF-related work disruptions.^59,60,61^ For policymakers, these findings may guide coverage and payment strategies that align resources with shifting care patterns while supporting quality and access.^70^ Future research should track long-term trajectories, including progression to permanent disability or early retirement, and evaluate the cost-effectiveness of emerging therapies. A fuller understanding of the societal burden of AF, including caregiver burden, job stability, and quality of life, will further guide public health and payer decisions.

### Limitations

This study has several limitations. First, the MarketScan Commercial Database captures individuals with employer-sponsored, noncapitated insurance—primarily from large firms—resulting in a population with only 38.7% females and likely underrepresenting lower-income, part-time, or precariously employed individuals and may understate burden in more vulnerable groups.^71^ Absence and disability fields are recorded only for employers participating in the HPM module, so productivity outcomes reflect that subset; because denominators differ by outcomes, cross-outcome contrasts should be interpreted as within-outcome AF vs non-AF differences. Second, some comorbidities in our adjustment set (eg, hemiplegia and paraplegia) may lie on the causal pathway; adjusting for these potential mediators could attenuate observed differences, although these conditions may also predate AF diagnosis and reflect baseline clinical severity. Third, administrative claims capture only billed encounters and may miss undiagnosed or untreated AF cases; important social determinants of health (eg, race and ethnicity, income, and education) are unavailable, leaving room for residual confounding.^72^ Fourth, AF subtype (paroxysmal, persistent, and permanent), incident vs prevalent AF, or catheter ablation were not reliably captured, precluding assessment of heterogeneity in costs and productivity losses. Fifth, productivity loss were limited to absenteeism and disability among employees with employer-sponsored benefits and did not include presenteeism, early retirement, or nonmarket productivity loss, making productivity loss estimates conservative. Sixth, the cross-sectional design precludes causal inference, despite extensive covariate adjustment and propensity-score overlap weighting.

## Conclusions

This cross-sectional study found that among privately insured employees aged 18 to 64 years in the US, persons with AF incurred $11 393 in excess annual medical costs per person compared with those without AF, primarily due to outpatient care. Productivity losses from sick leave and short-term disability added $840 per person annually. Females incurred higher AF-related emergency and inpatient care costs than males. These findings support strategies to improve outpatient access, strengthen chronic AF management, and reduce work-related disruptions for working-age adults.^32^

## Notes

Supplement 1. eTable 1. ICD-10-CM, DRG, and ICD-10-PCS procedure codes used for pregnancy exclusion eTable 2. ICD-10-CM diagnosis codes for atrial fibrillation, risk factors, and comorbidities eTable 3. Pairwise Correlations among AF status, comorbidities, and risk factors eTable 4. Summary table of variables included in iterative propensity score estimation method (N=1,612,398) eTable 5. Coefficients and odds ratio from logistic regression used to estimate propensity scores eTable 6. Summary of estimated propensity score from the logistic regression and weights calculated from the propensity scores eTable 7. Covariate balance between afib and non-afib population on the overlap weighted sample for all variables included in estimating propensity score and weights eFigure 1. Kernel density showing propensity score distribution of unweighted and weighted population using different weights eFigure 2. Overlap weighted distribution of propensity scores by sex eFigure 3. Overlap weighted distribution of propensity scores by age group eFigure 4. Overlap weighted distribution of propensity scores by urbanicity eTable 8. Adjusted medical costs and productivity losses associated with atrial fibrillation, GLM and negative binomial model eTable 9. Adjusted medical costs and productivity losses associated with atrial fibrillation without weighting eTable 10. Adjusted medical costs and productivity losses associated with atrial fibrillation using IPW eTable 11. Adjusted medical costs and productivity losses associated with atrial fibrillation under ATT-targeted weights eTable 12. Adjusted differences in medical costs and productivity losses among individuals with atrial fibrillation, stratified by sex eTable 13. Adjusted differences in medical costs and productivity losses among individuals with atrial fibrillation, stratified by rurality

Supplement 2. Data Sharing Statement
