# Supplementary material for: Medical Costs and Productivity Losses of Atrial Fibrillation Among US Privately Insured Employees
Source: JAMA Netw Open. 2026 Feb 12;9(2):e2559227. doi: 10.1001/jamanetworkopen.2025.59227 (PMC12902891; doi:10.1001/jamanetworkopen.2025.59227)
Supplement: Supplement 1. — eTable 1. ICD-10-CM, DRG, and ICD-10-PCS procedure codes used for pregnancy exclusion eTable 2. ICD-10-CM diagnosis codes for atrial fibrillation, risk factors, and comorbidities eTable 3. Pairwise Correlations among AF status, comorbidities, and risk factors eTable 4. Summary table of variables included in iterative propensity score estimation method (N=1,612,398) eTable 5. Coefficients and odds ratio from logistic regression used to estimate propensity scores eTable 6. Summary of estimated propensity score from the logistic regression and weights calculated from the propensity scores eTable 7. Covariate balance between afib and non-afib population on the overlap weighted sample for all variables included in estimating propensity score and weights eFigure 1. Kernel density showing propensity score distribution of unweighted and weighted population using different weights eFigure 2. Overlap weighted distribution of propensity scores by sex eFigure 3. Overlap weighted distribution of propensity scores by age group eFigure 4. Overlap weighted distribution of propensity scores by urbanicity eTable 8. Adjusted medical costs and productivity losses associated with atrial fibrillation, GLM and negative binomial model eTable 9. Adjusted medical costs and productivity losses associated with atrial fibrillation without weighting eTable 10. Adjusted medical costs and productivity losses associated with atrial fibrillation using IPW eTable 11. Adjusted medical costs and productivity losses associated with atrial fibrillation under ATT-targeted weights eTable 12. Adjusted differences in medical costs and productivity losses among individuals with atrial fibrillation, stratified by sex eTable 13. Adjusted differences in medical costs and productivity losses among individuals with atrial fibrillation, stratified by rurality [file jamanetwopen-e2559227-s001.pdf]

Supplemental Online Content

Zhang H, Lee JS, Kim S, et al. Medical costs, productivity losses, and atrial fibrillation among us privately insured employees. *JAMA Netw. Open*. 2026;9(2): e2559227. doi:10.1001/jamanetworkopen.2025.59227

eTable 1. *ICD-10-CM*, DRG, and *ICD-10-PCS* procedure codes used for pregnancy exclusion
eTable 2. *ICD-10-CM* diagnosis codes for atrial fibrillation, risk factors, and comorbidities
eTable 3. Pairwise Correlations among AF status, comorbidities, and risk factors
eTable 4. Summary table of variables included in iterative propensity score estimation method (N=1,612,398)
eTable 5. Coefficients and odds ratio from logistic regression used to estimate propensity scores
eTable 6. Summary of estimated propensity score from the logistic regression and weights calculated from the propensity scores
eTable 7. Covariate balance between afib and non-afib population on the overlap weighted sample for all variables included in estimating propensity score and weights
eFigure 1. Kernel density showing propensity score distribution of unweighted and weighted population using different weights
eFigure 2. Overlap weighted distribution of propensity scores by sex
eFigure 3. Overlap weighted distribution of propensity scores by age group
eFigure 4. Overlap weighted distribution of propensity scores by urbanicity
eTable 8. Adjusted medical costs and productivity losses associated with atrial fibrillation, glm and negative binomial model
eTable 9. Adjusted medical costs and productivity losses associated with atrial fibrillation without weighting
eTable 10. Adjusted medical costs and productivity losses associated with atrial fibrillation using IPW
eTable 11. Adjusted medical costs and productivity losses associated with atrial fibrillation under att-targeted weights
eTable 12. Adjusted differences in medical costs and productivity losses among individuals with atrial fibrillation, stratified by sex
eTable 13. Adjusted differences in medical costs and productivity losses among individuals with atrial fibrillation, stratified by rurality

This supplemental material has been provided by the authors to give readers additional information about their work.

© 2026 Zhang H et al. *JAMA Network Open*.

eTable 1. *ICD-10-CM*, DRG, and *ICD-10-PCS* procedure codes used for pregnancy exclusion

	ICD-10-CM	DRG	ICD-10-PCS Procedure
Pregnancy	O00-O99, O9A1-O9A5, Z33, Z34, Z36, Z37, Z3201, Z322, Z39, F53, A34	765, 766, 767, 768, 769, 770, 771, 772, 773, 775, 776, 777, 779, 780, 781, 782	10A0, 10D00Z0, 10D00Z1, 10D00Z2, 10D07Z3, 10D07Z4, 10D07Z5, 10D07Z6, 10D07Z7, 10D07Z8, 10E0XZZ

Abbreviations: CM, clinical modification; DRG, diagnosis-related group; ICD, International Classification of Diseases; PCS, procedure coding system.

© 2026 Zhang H et al. *JAMA Network Open*.

eTable 2. ***ICD-10-CM*** **diagnosis codes for atrial fibrillation, risk factors, and comorbidities**

	**ICD-10-CM**
**Atrial fibrillation**	I48
**Risk factors**	
1. Alcohol use	F10.10, F10.120, F10.121, F10.129, F10.14, F10.150, F10.151, F10.159, F10.180, F10.181, F10.182, F10.188, F10.19, F10.20, F10.220, F10.221, F10.229, F10.230, F10.231, F10.232, F10.239, F10.24, F10.250, F10.251, F10.259, F10.26, F10.27, F10.280, F10.281, F10.282, F10.288, F10.29, F10.920, F10.921, F10.929, F10.94, F10.950, F10.951, F10.959, F10.96, F10.97, F10.980, F10.981, F10.982, F10.988, F10.99, G62.1, I42.6, K29.20, K29.21, K70.0, K70.10, K70.11, K70.2, K70.30, K70.31, K70.40, K70.41, K70.9, P04.3, Q86.0, T51.0X1A, T51.0X2A, T51.0X3A, T51.0X4A, Z71.41, Z71.42
2. Tobacco use	F17.200, F17.201, F17.203, F17.208, F17.209, F17.210, F17.211, F17.213, F17.218, F17.219, F17.220, F17.221, F17.223, F17.228, F17.229, F17.290, F17.291, F17.293, F17.298, F17.299, O99.330, O99.331, O99.332, O99.333, O99.334, O99.335, T65.211A, T65.212A, T65.213A, T65.214A, T65.221A, T65.222A, T65.223A, T65.224A, T65.291A, T65.292A, T65.293A, T65.294A,Z72.0
3. Obesity	E66.01, E66.09, E66.1, E66.2, E66.8, E66.9, Z68.30, Z68.31, Z68.32, Z68.33, Z68.34, Z68.35, Z68.36, Z68.37, Z68.38, Z68.39, Z68.41, Z68.42, Z68.43, Z68.44, Z68.45
**Comorbidities**	
4. Hypertension	I10-I15
5. Lipid disorders (hyperlipidemia)	E78.0, E78.00, E78.01, E78.1, E78.2, E78.3, E78.4, E78.41, E78.49, E78.5
6. Myocardial infarction	I21.x, I22.x, I25.2
7. Congestive heart failure	I09.9, I11.0, I13.0, I13.2, I25.5, I42.0, I42.5-I42.9, I43.x, I50.x, P29.0
8. Peripheral vascular	I70.x, I71.x, I73.1, I73.8, I73.9, I77.1, I79.0, I79.2, K55.1, K55.8, K55.9, Z95.8, Z95.9
9. Chronic pulmonary disease	I27.8, I27.9, J40.x-J47.x, J60.x-J67.x, J68.4, J70.1, J70.3
10. Rheumatic disease	M05.x, M06.x, M31.5, M32.x-M34.x, M35.1, M35.3, M36.0
11. Peptic ulcer disease	K25.x-K28.x
12. Diabetes	E10.x-E14.x
13. Hemiplegia or paraplegia	G04.1, G11.4, G80.1, G80.2, G81.x, G82.x, G83.0-G83.4, G83.9

© 2026 Zhang H et al. *JAMA Network Open*.

14.	Renal disease	I12.0, I13.1, N03.2-N03.7, N05.2-N05.7, N18.x, N19.x, N25.0, Z49.0-Z49.2, Z94.0, Z99.2
15.	Malignancy	C00.x-C26.x, C30.x-C34.x, C37.x-C41.x, C43.x, C45.x-C58.x, C60.x-C76.x, C81.x-C85.x, C88.x, C90.x-C97.x
16.	Liver disease	B18.x, I85.0, I85.9, I86.4, I98.2, K70.0-K70.4, K70.9, K71.1, K71.3-K71.5, K71.7, K72.1, K72.9, , K73.x, K74.x, K76.0, K76.2-K76.4, K76.5, K76.6, K76.7, K76.8, K76.9, Z94.4
17.	HIV/AIDS	B20.x-B22.x, B24.x
18.	COVID-19 infection	U07.1

Source:

1. Centers for Medicare & Medicaid Services. Chronic Condition Warehouse: Condition Categories - Chronic. Published 2024. https://www2.ccwdata.org/web/guest/condition-categories-chronic
2. Quan H, Sundararajan V, Halfon P, et al. Coding algorithms for defining comorbidities in ICD-9-CM and ICD-10 administrative data. Med Care. 2005;43(11):1130–1139. https://journals.lww.com/lww-medicalcare/abstract/2005/11000/coding_algorithms_for_defining_comorbidities_in.10.aspx

© 2026 Zhang H et al. *JAMA Network Open*.

eTable 3: Pairwise Correlations among AF Status, Comorbidities, and Risk Factors

	Afib	1	2	3	4	5	6	7	8	9	10	11	12	13	14	15	16	17	18
1	**0.2**	1.0																	
2	**0.2**	**0.5**	1.0																
3	**0.3**	**0.1**	**0.3**	1.0															
4	**0.5**	**0.3**	**0.3**	**0.5**	**1.0**														
5	**0.4**	**0.1**	**0.3**	**0.4**	**0.7**	**1.0**													
6	**0.4**	**0.2**	**0.4**	**0.3**	**0.5**	**0.5**	**1.0**												
7	**0.7**	**0.3**	**0.3**	**0.4**	**0.6**	**0.4**	**0.7**	**1.0**											
8	**0.5**	**0.2**	**0.4**	**0.3**	**0.5**	**0.4**	**0.5**	**0.7**	**1.0**										
9	**0.2**	**0.2**	**0.4**	**0.3**	**0.3**	**0.2**	**0.2**	**0.3**	**0.3**	**1.0**									
10	**0.1**	0.0	**0.1**	**0.2**	**0.2**	**0.1**	**0.1**	**0.1**	**0.1**	**0.2**	**1.0**								
11	**0.2**	**0.4**	**0.3**	**0.2**	**0.3**	**0.2**	**0.3**	**0.3**	**0.3**	**0.2**	**0.2**	**1.0**							
12	**0.3**	**0.1**	**0.2**	**0.4**	**0.6**	**0.7**	**0.3**	**0.4**	**0.3**	**0.2**	**0.1**	**0.2**	**1.0**						
13	**0.3**	**0.3**	**0.3**	**0.2**	**0.4**	**0.3**	**0.3**	**0.4**	**0.4**	**0.2**	**0.1**	**0.3**	**0.3**	**1.0**					
14	**0.3**	**0.2**	**0.2**	**0.3**	**0.6**	**0.4**	**0.4**	**0.5**	**0.4**	**0.2**	**0.2**	**0.3**	**0.4**	**0.3**	**1.0**				
15	**0.2**	**0.1**	**0.2**	**0.2**	**0.2**	**0.2**	**0.2**	**0.2**	**0.3**	**0.2**	**0.1**	**0.2**	**0.2**	**0.2**	**0.3**	**1.0**			
16	**0.2**	**0.5**	**0.3**	**0.3**	**0.3**	**0.3**	**0.2**	**0.3**	**0.3**	**0.2**	**0.2**	**0.4**	**0.3**	**0.2**	**0.3**	**0.3**	**1.0**		
17	0.0	**0.1**	**0.1**	**0.0**	**0.2**	**0.2**	**0.1**	**0.1**	**0.1**	**0.1**	**-0.1**	**0.1**	0.1	0.0	**0.3**	**0.1**	**0.2**	**1.0**	
18	**0.1**	**0.0**	**0.1**	**0.2**	**0.1**	**0.1**	**0.1**	**0.1**	**0.1**	**0.2**	**0.1**	**0.1**	**0.1**	**0.1**	**0.1**	**0.0**	**0.1**	**0.0**	**1.0**

Note: Condition numbers 1 to 18 are the same as reported in eTable2. Cells report correlation coefficients (ρ) estimated using pairwise deletion. For collinearity screening, we used |ρ|>.80; no pair exceeded this threshold. Boldface indicates 2-sided P<.05 (Fisher exact) and is included for descriptive completeness.

© 2026 Zhang H et al. *JAMA Network Open*.

eTable 4: Summary Table of Variables Included in Iterative Propensity Score Estimation Method (N=1,612,398)

Variable	Mean	Std. Dev.	Min	Max
Hypertension	.13	.34	0	1
Congestive heart failure	.01	.07	0	1
Age	44	11.11	17	64
Female	.39	.49	0	1
Obesity	.07	.25	0	1
Peripheral vascular	0	.07	0	1
Chronic pulmonary	.02	.15	0	1
Lipid disorders	.11	.31	0	1
Alcohol	.01	.07	0	1
COVID-19 infection	.09	.29	0	1
Diabetes	.06	.24	0	1
Hemiplegia or paraplegia	0	.03	0	1
Any malignancy	.02	.12	0	1
South	.43	.5	0	1
Liver disease	.01	.09	0	1
Myocardial infarction	.01	.07	0	1
Urban	.92	.27	0	1
Tobacco	.01	.12	0	1
HIV/AIDS	0	.05	0	1
Rheumatic disease	.01	.08	0	1
Peptic ulcer disease	0	.03	0	1
Interaction of hypertension and lipid disorders	.06	.23	0	1
Interaction of hypertension and congenital heart failure	0	.06	0	1
Interaction of congenital heart failure and myocardial infarction	0	.03	0	1
Interaction of congenital heart failure and peripheral vascular	0	.03	0	1
Interaction of age and peripheral vascular	.25	3.68	0	64
Interaction of congenital heart failure and lipid disorder	0	.05	0	1
Interaction of peripheral vascular and tobacco	0	.02	0	1
Interaction of hypertension and obesity	.03	.17	0	1
Interaction of hypertension and peripheral vascular	0	.05	0	1
Interaction of congenital heart failure and chronic pulmonary	0	.02	0	1
Interaction of hypertension and age	6.84	17.69	0	64
Interaction of congenital heart failure and any malignancy	0	.02	0	1
Interaction of age and age	2058.97	970.18	289	4096
Interaction of lipid disorder and myocardial infarction	0	.05	0	1
Interaction of hypertension and tobacco	.01	.07	0	1
Interaction of congenital heart failure and obesity	0	.04	0	1
Interaction of age and tobacco	.65	5.61	0	64
Interaction of female and myocardial infarction	0	.03	0	1
Interaction of alcohol and peptic ulcer	0	.01	0	1
Interaction of obesity and lipid disorder	.02	.14	0	1
Interaction of age and myocardial infarction	.27	3.81	0	64
Interaction of obesity and any malignancy	0	.05	0	1
Interaction of peripheral vascular and lipid disorder	0	.05	0	1
Interaction of obesity and COVID-19 infection	.01	.1	0	1
Interaction of obesity and diabetes	.01	.12	0	1

© 2026 Zhang H et al. *JAMA Network Open*.

Interaction of peripheral vascular and urban	0	.07	0	1
Interaction of chronic pulmonary and lipid disorder	.01	.07	0	1
Interaction of congenital heart failure and tobacco	0	.02	0	1
Interaction of hypertension and hemiplegia or paraplegia	0	.02	0	1
Interaction of female and diabetes	.02	.14	0	1
Interaction of obesity and peripheral vascular	0	.03	0	1
Interaction of hemiplegia or paraplegia and any malignancy	0	.01	0	1
Interaction of hypertension and chronic pulmonary	.01	.08	0	1
Interaction of chronic pulmonary and diabetes	0	.05	0	1
Interaction of congenital heart failure and covid-19 infection	0	.03	0	1
Interaction of female and south region	.17	.37	0	1
Interaction of obesity and liver disease	0	.05	0	1
Interaction of diabetes and south region	.03	.17	0	1
Interaction of congenital heart failure and south region	0	.05	0	1
Interaction of age and lipid disorder	5.45	16.1	0	64
Interaction of obesity and chronic pulmonary	0	.07	0	1
Interaction of south region and peptic ulcer	0	.02	0	1
Interaction of congenital heart failure and age	.28	3.87	0	64
Interaction of lipid disorder and any malignancy	0	.06	0	1

© 2026 Zhang H et al. *JAMA Network Open*.

eTable 5: Coefficients and Odds Ratio from Logistic Regression Used to Estimate Propensity Scores

VARIABLES	(1)<br>**Coefficients**	(2)<br>**Odds ratio**
Hypertension	2.45	11.64
	(2.13 – 2.78)	(8.41 – 16.10)
Congestive heart failure	4.16	64.23
	(3.67 – 4.66)	(39.18 – 105.30)
Age	0.04	1.04
	(0.01 – 0.06)	(1.01 – 1.07)
Female	-0.83	0.44
	(-0.90 – -0.76)	(0.41 – 0.47)
Obesity	1.14	3.13
	(1.03 – 1.24)	(2.81 – 3.47)
Peripheral vascular	4.1	60.2
	(3.42 – 4.77)	(30.61 – 118.40)
Chronic pulmonary	0.79	2.20
	(0.63 – 0.95)	(1.88 – 2.58)
Lipid disorders	0.62	1.86
	(0.24 – 1.00)	(1.27 – 2.71)
Alcohol	0.61	1.84
	(0.47 – 0.75)	(1.60 – 2.12)
COVID-19 infection	0.32	1.37
	(0.24 – 0.39)	(1.27 – 1.48)
Diabetes	-0.24	0.79
	(-0.32 – -0.15)	(0.72 – 0.86)
Hemiplegia or paraplegia	1.67	5.29
	(1.16 – 2.17)	(3.19 – 8.79)
Any malignancy	0.45	1.57
	(0.32 – 0.59)	(1.37 – 1.81)
South region	-0.07	0.93
	(-0.13 – -0.02)	(0.88 – 0.98)
Liver disease	0.3	1.35
	(0.15 – 0.45)	(1.16 – 1.57)
Myocardial infarction	2.23	9.34
	(1.49 – 2.98)	(4.44 – 19.63)
Urban	-0.11	0.89
	(-0.18 – -0.04)	(0.83 – 0.96)
Tobacco	1.72	5.59
	(1.08 – 2.36)	(2.95 – 10.59)
HIV/AIDS	-0.51	0.6
	(-0.91 – -0.12)	(0.40 – 0.89)
Rheumatic disease	0.17	1.18
	(-0.01 – 0.35)	(0.99 – 1.42)
Peptic ulcer disease	0.06	1.06
	(-0.43 – 0.55)	(0.65 – 1.74)

© 2026 Zhang H et al. *JAMA Network Open*.

Interaction of hypertension and lipid disorders	-0.84<br>(-0.94 – -0.75)	0.43<br>(0.39 – 0.47)
Interaction of hypertension and congenital heart failure	-0.91<br>(-1.05 – -0.76)	0.4<br>(0.35 – 0.47)
Interaction of congenital heart failure and myocardial infarction	-1.26<br>(-1.44 – -1.07)	0.28<br>(0.24 – 0.34)
Interaction of congenital heart failure and peripheral vascular	-1.07<br>(-1.24 – -0.91)	0.34<br>(0.29 – 0.40)
Interaction of age and peripheral vascular	-0.04<br>(-0.05 – -0.03)	0.96<br>(0.95 – 0.97)
Interaction of congenital heart failure and lipid disorder	-0.4<br>(-0.53 – -0.28)	0.67<br>(0.59 – 0.76)
Interaction of peripheral vascular and tobacco	0.51<br>(-0.95 – -0.41)	(0.39 – 0.67)
Interaction of hypertension and obesity	-0.31<br>(-0.42 – -0.20)	0.73<br>(0.66 – 0.82)
Interaction of hypertension and peripheral vascular	-0.46<br>(-0.64 – -0.28)	0.63<br>(0.53 – 0.76)
Interaction of congenital heart failure and chronic pulmonary	-0.34<br>(-0.53 – -0.15)	0.71<br>(0.59 – 0.86)
Interaction of hypertension and age	-0.02<br>(-0.02 – -0.01)	0.98<br>(0.98 – 0.99)
Interaction of congenital heart failure and any malignancy	-0.51<br>(-0.77 – -0.26)	0.6<br>(0.46 – 0.77)
Interaction of age and age	0.00<br>(0.00 – 0.00)	1.00<br>(1.00 – 1.00)
Interaction of lipid disorder and myocardial infarction	-0.31<br>(-0.50 – -0.13)	0.73<br>(0.61 – 0.88)
Interaction of hypertension and tobacco	-0.33<br>(-0.54 – -0.11)	0.72<br>(0.59 – 0.89)
Interaction of congenital heart failure and obesity	-0.25<br>(-0.38 – -0.12)	0.78<br>(0.69 – 0.89)
Interaction of age and tobacco	-0.02<br>(-0.03 – -0.01)	0.98<br>(0.97 – 0.99)
Interaction of female and myocardial infarction	0.42<br>(0.19 – 0.65)	1.52<br>(1.21 – 1.92)
Interaction of alcohol and peptic ulcer	-2.68<br>(-4.73 – -0.64)	0.07<br>(0.01 – 0.53)
Interaction of obesity and lipid disorder	-0.17<br>(-0.27 – -0.07)	0.84<br>(0.76 – 0.93)
Interaction of age and myocardial infarction	-0.02<br>(-0.04 – -0.01)	0.98<br>(0.97 – 0.99)
Interaction of obesity and any malignancy	-0.26<br>(-0.47 – -0.05)	0.77<br>(0.62 – 0.95)
Interaction of peripheral vascular and lipid disorder	-0.23<br>(-0.39 – -0.07)	0.8<br>(0.68 – 0.94)
Interaction of obesity and COVID-19 infection	-0.16	0.85

© 2026 Zhang H et al. *JAMA Network Open*.

	(-0.29 – -0.03)	(0.75 – 0.97)
Interaction of obesity and diabetes	0.14	1.15
	(0.03 – 0.26)	(1.03 – 1.29)
Interaction of peripheral vascular and urban	0.42	1.52
	(0.13 – 0.70)	(1.14 – 2.02)
Interaction of chronic pulmonary and lipid disorder	-0.24	0.79
	(-0.39 – -0.08)	(0.68 – 0.92)
Interaction of congenital heart failure and tobacco	-0.35	0.7
	(-0.59 – -0.12)	(0.56 – 0.89)
Interaction of hypertension and hemiplegia or paraplegia	-0.83	0.44
	(-1.39 – -0.27)	(0.25 – 0.77)
Interaction of female and diabetes	0.17	1.18
	(0.04 – 0.30)	(1.04 – 1.34)
Interaction of obesity and peripheral vascular	-0.2	0.82
	(-0.37 – -0.03)	(0.69 – 0.98)
Interaction of hemiplegia or paraplegia and any malignancy	-1.08	0.34
	(-2.07 – -0.08)	(0.13 – 0.93)
Interaction of hypertension and chronic pulmonary	-0.22	0.81
	(-0.39 – -0.04)	(0.68 – 0.96)
Interaction of chronic pulmonary and diabetes	0.24	1.27
	(0.05 – 0.42)	(1.05 – 1.53)
Interaction of congenital heart failure and COVID-19 infection	-0.2	0.82
	(-0.36 – -0.03)	(0.70 – 0.97)
Interaction of female and south region	-0.11	0.89
	(-0.22 – -0.01)	(0.81 – 0.99)
Interaction of obesity and liver disease	-0.25	0.78
	(-0.48 – -0.01)	(0.62 – 0.99)
Interaction of diabetes and south region	-0.13	0.88
	(-0.24 – -0.03)	(0.79 – 0.97)
Interaction of congenital heart failure and south region	0.14	1.14
	(0.02 – 0.25)	(1.02 – 1.29)
Interaction of age and lipid disorder	0.01	1.01
	(0.00 – 0.01)	(1.00 – 1.02)
Interaction of obesity and chronic pulmonary	-0.17	0.85
	(-0.33 – -0.01)	(0.72 – 0.99)
Interaction of south region and peptic ulcer	0.67	1.95
	(0.02 – 1.32)	(1.02 – 3.76)
Interaction of congenital heart failure and age	-0.01	0.99
	(-0.02 – 0.00)	(0.98 – 1.00)
Interaction of lipid disorder and any malignancy	-0.19	0.83
	(-0.38 – 0.00)	(0.69 – 1.00)
Observations	1,612,398	1,612,398

© 2026 Zhang H et al. *JAMA Network Open*.

eTable 6. Summary of Estimated Propensity Score from the Logistic Regression and Weights Calculated from the Propensity Scores

	Observation	Mean	Std. Dev.	Median	Min	Max
**Full Sample**						
Propensity score						
Total	1612398	0.00632	0.020947	0.00153	0.0000636	0.767509
Adults without Afib	1602208	0.005903	0.018435	0.001512	0.0000636	0.767509
Adults with Afib	10190	0.071829	0.10807	0.026221	0.0001249	0.68375
Overlap weights						
Total	1612398	0.011732	0.075849	0.00153	0.0000636	0.999875
Adults without Afib	1602208	0.005903	0.018435	0.001512	0.0000636	0.767509
Adults with Afib	10190	0.928171	0.10807	0.973779	0.31625	0.999875
Inverse probability weights						
Total	1612398	1.914396	31.58902	1.001532	1.000064	8009.3
Adults without Afib	1602208	1.00641	0.02624	1.001515	1.000064	4.301246
Adults with Afib	10190	144.6801	370.6715	38.13676	1.462523	8009.3
Average treatment effect on the treated weights						
Total	1612398	0.0126895	0.0829685	0.0015319	0.0000636	3.301246
Adults without Afib	1602208	0.0064102	0.0262399	0.0015145	0.0000636	3.301246
Adults with Afib	10190	1	0	1	1	1

Note: This table presents summary statistics for estimated propensity scores and associated weights under three weighting approaches: overlap weighting (OW), inverse probability weighting (IPW), and average treatment effect on the treated (ATT) weighting. OW assign greater weight to individuals with the highest covariate overlap between groups, minimizing extrapolation and extreme values. IPW assigns weights inverse to the estimated probability of treatment, often leading to large weights for rare cases and greater variability. ATT weighting reweights the control group to resemble the distribution of covariates in the treated group. Among the three, OW yielded weights with tighter distributions, avoided extreme values, and showed better covariate balance between AF and non-AF individuals (see eFigure 6 and eFigures 1-4). Based on these statistical advantages, OW was selected for the main analysis.

© 2026 Zhang H et al. *JAMA Network Open*.

eTable 7. Covariate Balance between Afib and Non-Afib Population on the Overlap Weighted Sample for All Variables Included in Estimating Propensity Score and Weights

	Unweighted			Weighted		
	Adults with Afib	Adults without Afib	Std diff.	Adults with Afib	Adults without Afib	Std. diff.
Hypertension	0.60	0.13	1.12	0.59	0.59	0
Congestive heart failure	0.18	0.00	0.64	0.14	0.14	0
Age	54.28	43.99	1.08	54.1	54.1	0
Female	0.20	0.39	-0.41	0.21	0.21	0
Obesity	0.27	0.07	0.56	0.26	0.26	0
Peripheral vascular	0.10	0.00	0.43	0.08	0.08	0
Chronic pulmonary	0.08	0.02	0.28	0.08	0.08	0
Lipid disorders	0.41	0.10	0.75	0.4	0.4	0
Alcohol	0.03	0.01	0.17	0.02	0.02	0
COVID-19 infection	0.14	0.09	0.15	0.14	0.14	0
Diabetes	0.20	0.06	0.43	0.19	0.19	0
Hemiplegia or paraplegia	0.01	0.00	0.13	0.01	0.01	0
Any malignancy	0.05	0.02	0.20	0.05	0.05	0
South region	0.42	0.43	-0.01	0.42	0.42	0
Liver disease	0.04	0.01	0.19	0.03	0.03	0
Myocardial infarction	0.06	0.00	0.33	0.06	0.06	0
Urban	0.90	0.92	-0.07	0.9	0.9	0
Tobacco	0.05	0.01	0.22	0.05	0.05	0
HIV/AIDS	0.00	0.00	0.01	0	0	0
Rheumatic disease	0.01	0.01	0.07	0.01	0.01	0
Peptic ulcer disease	0.00	0.00	0.07	0	0	0
Interaction of hypertension and lipid disorders	0.31	0.06	0.70	0.3	0.3	0
Interaction of hypertension and congenital heart failure	0.14	0.00	0.54	0.11	0.11	0
Interaction of congenital heart failure and myocardial infarction	0.03	0.00	0.24	0.03	0.03	0
Interaction of congenital heart failure and peripheral vascular	0.05	0.00	0.30	0.03	0.03	0
Interaction of age and peripheral vascular	5.25	0.22	0.43	4.47	4.47	0
Interaction of congenital heart failure and lipid disorder	0.08	0.00	0.41	0.07	0.07	0
Interaction of peripheral vascular and tobacco	0.01	0.00	0.12	0.01	0.01	0
Interaction of hypertension and obesity	0.21	0.03	0.58	0.2	0.2	0
Interaction of hypertension and peripheral vascular	0.07	0.00	0.36	0.06	0.06	0
Interaction of congenital heart failure and chronic pulmonary	0.02	0.00	0.21	0.02	0.02	0
Interaction of hypertension and age	33.30	6.69	1.15	32.38	32.38	0
Interaction of congenital heart failure and any malignancy	0.01	0.00	0.14	0.01	0.01	0
Interaction of age and age	3005.20	2057.28	1.09	2986.75	2986.75	0
Interaction of lipid disorder and myocardial infarction	0.04	0.00	0.26	0.04	0.04	0
Interaction of hypertension and tobacco	0.04	0.01	0.23	0.04	0.04	0

© 2026 Zhang H et al. *JAMA Network Open*.

Interaction of congenital heart failure and obesity	0.07	0.00	0.37	0.05	0.05	0
Interaction of age and tobacco	2.79	0.63	0.23	2.7	2.7	0
Interaction of female and myocardial infarction	0.01	0.00	0.12	0.01	0.01	0
Interaction of alcohol and peptic ulcer	0.00	0.00	0.00	0	0	0
Interaction of obesity and lipid disorder	0.13	0.02	0.44	0.13	0.13	0
Interaction of age and myocardial infarction	3.56	0.25	0.33	3.27	3.27	0
Interaction of obesity and any malignancy	0.01	0.00	0.13	0.01	0.01	0
Interaction of peripheral vascular and lipid disorder	0.05	0.00	0.29	0.04	0.04	0
Interaction of obesity and COVID-19 infection	0.05	0.01	0.22	0.04	0.04	0
Interaction of obesity and diabetes	0.09	0.01	0.34	0.08	0.08	0
Interaction of peripheral vascular and urban	0.09	0.00	0.41	0.08	0.08	0
Interaction of chronic pulmonary and lipid disorder	0.04	0.00	0.23	0.03	0.03	0
Interaction of congenital heart failure and tobacco	0.01	0.00	0.16	0.01	0.01	0
Interaction of hypertension and hemiplegia or paraplegia	0.01	0.00	0.12	0.01	0.01	0
Interaction of female and diabetes	0.04	0.02	0.11	0.04	0.04	0
Interaction of obesity and peripheral vascular	0.03	0.00	0.23	0.02	0.02	0
Interaction of hemiplegia or paraplegia and any malignancy	0.00	0.00	0.03	0	0	0
Interaction of hypertension and chronic pulmonary	0.06	0.01	0.30	0.06	0.06	0
Interaction of chronic pulmonary and diabetes	0.02	0.00	0.18	0.02	0.02	0
Interaction of congenital heart failure and COVID-19 infection	0.03	0.00	0.24	0.02	0.02	0
Interaction of female and south region	0.09	0.17	-0.24	0.09	0.09	0
Interaction of obesity and liver disease	0.01	0.00	0.14	0.01	0.01	0
Interaction of diabetes and south region	0.09	0.03	0.25	0.08	0.08	0
Interaction of congenital heart failure and south region	0.08	0.00	0.40	0.07	0.07	0
Interaction of age and lipid disorder	22.97	5.35	0.78	22.6	22.6	0
Interaction of obesity and chronic pulmonary	0.03	0.00	0.21	0.03	0.03	0
Interaction of south region and peptic ulcer	0.00	0.00	0.06	0	0	0
Interaction of congenital heart failure and age	9.89	0.22	0.63	7.79	7.79	0
Interaction of lipid disorder and any malignancy	0.02	0.00	0.16	0.02	0.02	0

© 2026 Zhang H et al. *JAMA Network Open*.

eFigure 1: Kernel density showing propensity score distribution of unweighted and weighted population using different weights

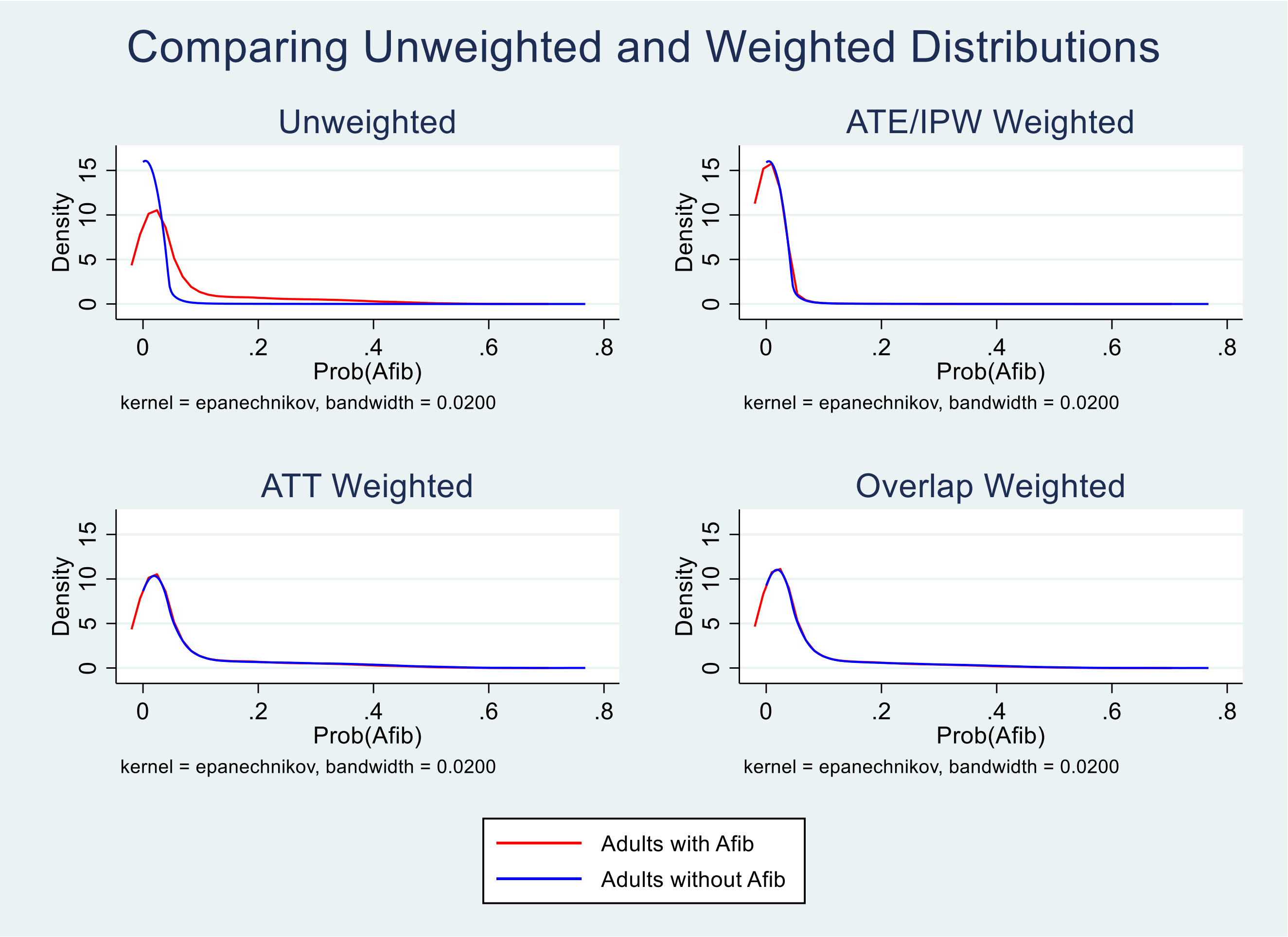

Abbreviations: ATE, average treatment effect (across the entire population); IPW, inverse probability weighting; ATT, average treatment effect on the treated.

© 2026 Zhang H et al. *JAMA Network Open*.

eFigure 2: Overlap Weighted Distribution of Propensity Scores by Sex

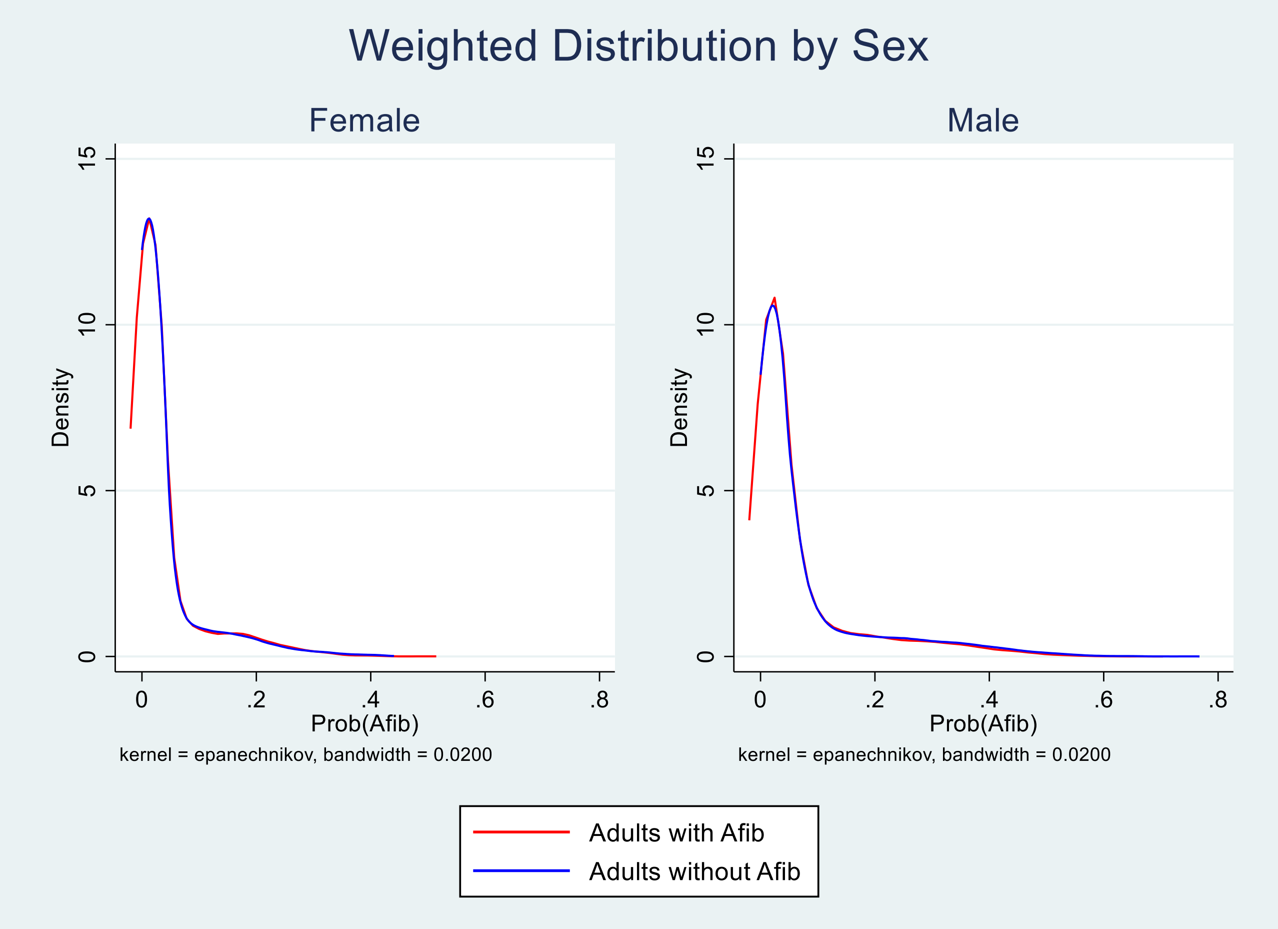

© 2026 Zhang H et al. *JAMA Network Open*.

eFigure 3: Overlap Weighted Distribution of Propensity Scores by Age Group

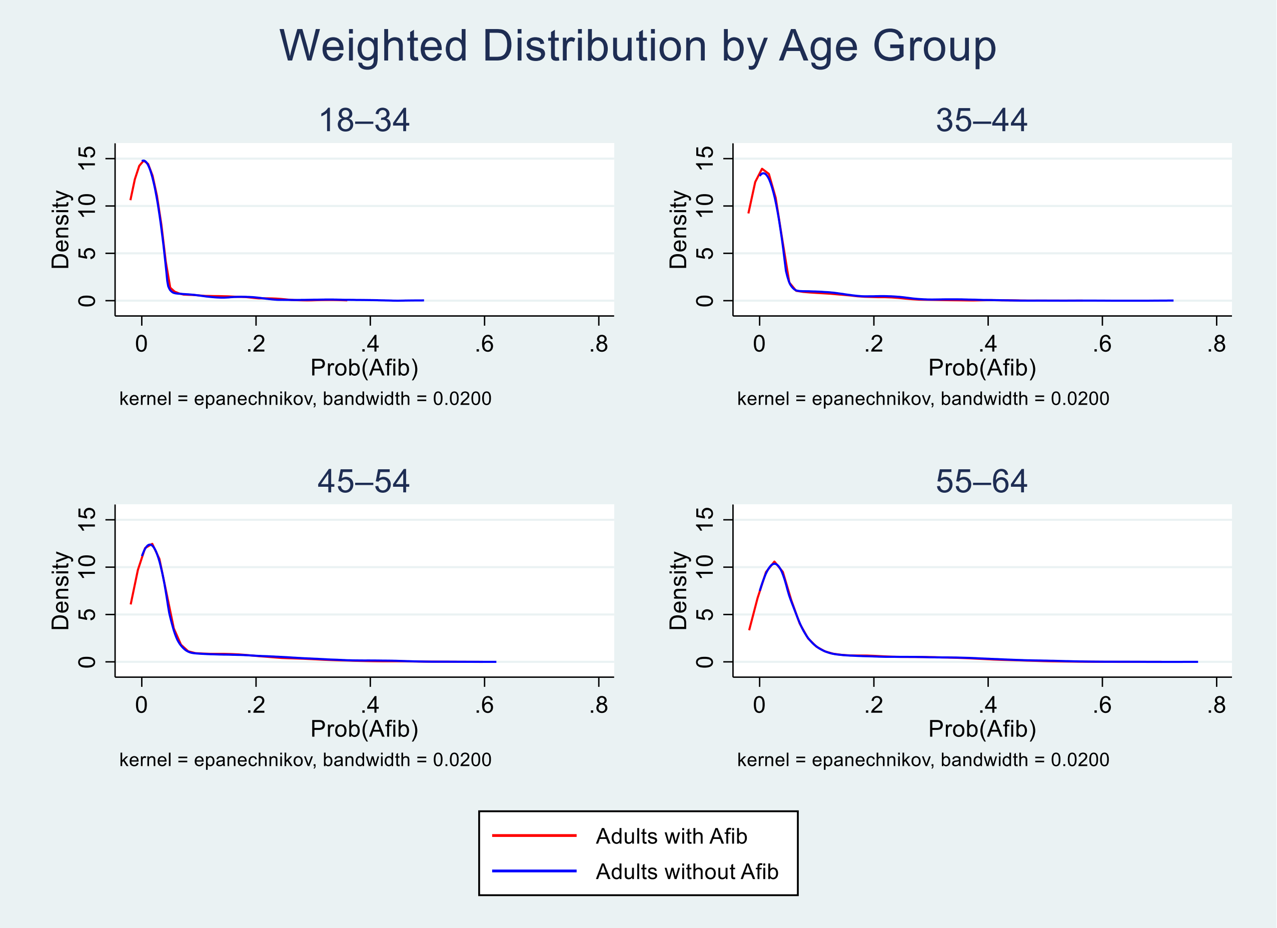

© 2026 Zhang H et al. *JAMA Network Open*.

eFigure 4: Overlap Weighted Distribution of Propensity Scores by Urbanicity

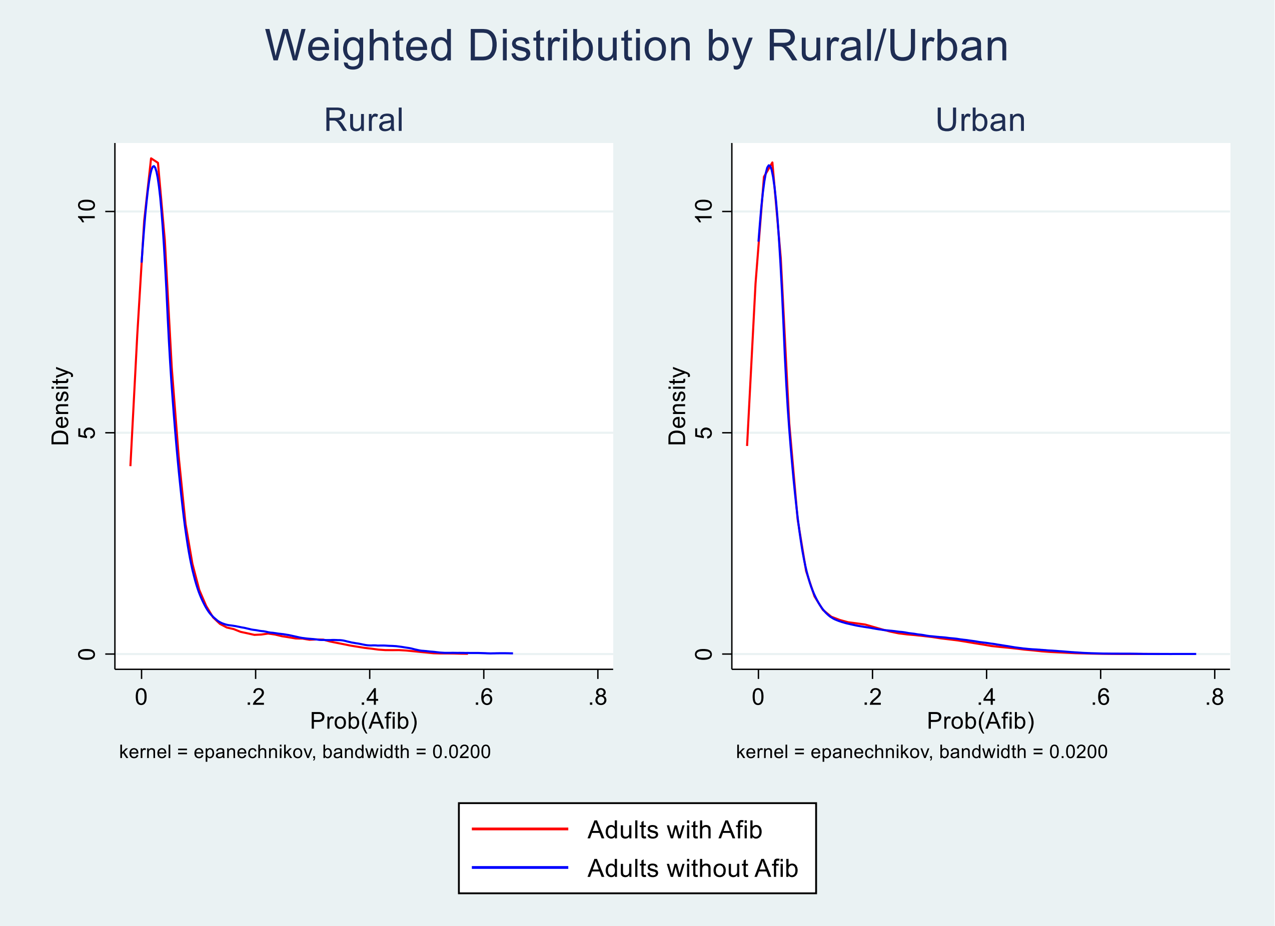

© 2026 Zhang H et al. *JAMA Network Open*.

eTable 8. Adjusted Medical costs and Productivity Losses Associated with Atrial Fibrillation, GLM and Negative Binomial Model[a]

Category	Without AF (95% CI)	With AF (95% CI)	Difference [With AF-Without AF] (95% CI)	p-value	N
**Panel A. Medical Costs[b]**					
Total Medical Costs, $	28,412.03 (27,575.92–29,248.14)	68,142.77 (65,551.64–70,733.92)	39,730.74 (37,501.38–41,960.12)	<.001	1,612,398
ED costs, $	1,052.09 (1,018.66–1,085.53)	2,393.30 (2,244.85–2,541.75)	1,341.20 (1,202.40–1,480.01)	<.001	1,612,398
Inpatient costs, $	32,561.03 (25,147.24–39,974.81)	115172.13 (93,891.16–136453.06)	82,611.10 (67,578.34–97,643.86)	<.001	1,612,398
Outpatient costs, $	9,679.35 (9,463.10–9,895.60)	23,961.66 (23,040.06–24,883.26)	14,282.31 (13,405.93–15,158.68)	<.001	1,612,398
Pharmacy costs, $	4,895.75 (4,719.75–5,071.76)	8,737.59 (8,195.67–9,279.52)	3,841.84 (3,330.85–4,352.83)	<.001	1,612,398
Number of ED visits	0.38 (0.37–0.39)	0.73 (0.70–0.76)	0.34 (0.31–0.37)	<.001	1,612,398
Number of inpatient admissions	0.22 (0.21–0.22)	0.41 (0.40–0.43)	0.20 (0.18–0.22)	<.001	1,612,398
Number of outpatient visits	16.43 (16.31–16.55)	24.03 (23.63–24.43)	7.59 (7.20–7.98)	<.001	1,612,398
Number of pharmacy prescriptions	23.99 (23.87–24.12)	32.89 (32.45–33.33)	8.90 (8.44–9.35)	<.001	1,612,398
**Panel B. Productivity Loss[c]**					
Num. of sick leave	4.82 (4.23–5.42)	5.79 (4.74–6.84)	0.91 (0.02–1.93)	0.07	139,490
Num. of short-term disability	8.98 (8.73–9.23)	11.91 (11.15–12.67)	4.91 (2.14–3.72)	<.001	1,355,064
Num. of long-term disability	2.17 (1.94–2.39)	2.57 (2.01–3.14)	0.53 (–0.20–1.02)	0.51	1,331,963
Sick leave payment, $	1,340.73 (1,176.61–1,507.63)	1,610.55 (1,318.48–1,902.61)	253.12 (5.57–536.85)	0.07	139,490
Short-term disability payment, $	1,748.52 (1,699.83–1,797.19)	2,319.01 (2,171.04–2,467.00)	956.03 (416.68–724.33)	<.001	1,355,064
Long-term disability payment, $	362.16 (323.78–398.88)	428.92 (335.47–524.05)	88.45 (-33.38–170.24)	0.51	1,331,963

Abbreviations: AF, atrial fibrillation; SD, standard deviation; ED, emergency department; GLM, Generalized Linear Model.

[a] Outcomes are all-cause and reported per person-year. AF and non-AF groups were balanced using propensity-score overlap weighting. Count outcomes (health care utilization; days of sick leave, short-term disability, long-term disability) were modeled with negative binomial regressions. Models adjusted for age, sex, urbanicity, census region, risk factors, and comorbidities. We report adjusted means (95% CIs) by AF status and the adjusted mean difference (AF–non-AF). Wilcoxon nonparametric rank-sum tests was used to test the differences in means for continuous variables by AF diagnosis status.

[b] Medical costs were estimated GLMs and were converted to 2024 USD using the Personal Consumption Expenditures Health Price Index from the U.S. Bureau of Economic Analysis. Because total and component costs were estimated separately with nonlinear links, and laboratory tests were not reported as a standalone category, components may not sum exactly to totals.

[c] Dollar values for productivity losses were calculated as: days × 8 hours × the 2021 average hourly wage for private nonfarm employees (U.S. Bureau of Labor Statistics), valuing sick leave at 100% and applying wage-replacement factors of 70% (short-term disability) and 60% (long-term disability). Values were converted to 2024 USD using the Employment Cost Index (Total Compensation for Civilian Workers) from the U.S. Bureau of Labor Statistics.

© 2026 Zhang H et al. *JAMA Network Open*.

eTable 9. Adjusted Medical Costs and Productivity Losses Associated with Atrial Fibrillation without Weighting[a]

Category	Without AF (95% CI)	With AF (95% CI)	Difference [With AF-Without AF] (95% CI)	p-value	N
**Panel A. Medical Costs[b]**					
**Total Medical Costs, $**	5,996.17 (5,967.91–6,024.43)	15,711.03 (15,101.08–16,320.98)	9,714.87 (9,104.77–10,324.96)	<.001	1,612,398
ED costs, $	527.16 (523.18–531.13)	1,135.45 (1,063.62–1,207.28)	608.29 (536.26–680.32)	<.001	1,612,398
Inpatient costs, $	1,355.00 (1,329.75–1,380.23)	3,598.91 (3,323.13–3,874.69)	2,243.92 (1,964.56–2,523.28)	<.001	1,612,398
Outpatient costs, $	-	-	-	-	-
Pharmacy costs, $	2,499.55 (2,465.68–2,533.42)	4,696.77 (4,358.59–5,034.94)	2,197.21 (1,859.82–2,534.61)	<.001	1,612,398
Number of ED visits	0.20 (0.20–0.20)	0.51 (0.49–0.53)	0.31 (0.29–0.33)	<.001	1,612,398
Number of inpatient admissions	0.03 (0.03–0.03)	0.12 (0.11–0.12)	0.08 (0.07–0.09)	<.001	1,612,398
Number of outpatient visits	-	-	-	-	-
Number of pharmacy prescriptions	11.94 (11.92–11.96)	17.00 (16.71–17.28)	5.06 (4.77–5.35)	<.001	1,612,398
**Panel B. Productivity Loss[c]**					
Num. of sick leave	2.39 (2.34–2.44)	3.09 (2.51–3.67)	0.71 (0.12–1.29)	0.02	139,490
Num. of short-term disability	3.13 (3.10–3.16)	4.43 (4.08–4.77)	1.30 (0.95–1.65)	<.001	1,355,064
Num. of long-term disability	0.45 (0.44–0.47)	0.56 (0.42–0.70)	0.11 (–0.04–0.25)	0.14	1,331,963
Sick leave payment, $	664.80 (650.89–678.71)	859.51 (698.18–1,020.85)	197.49 (33.38–358.83)	0.02	139,490
Short-term disability payment, $	609.45 (603.61–615.28)	862.57 (794.43–928.77)	253.12 (184.98–321.28)	<.001	1,355,064
Long-term disability payment, $	75.11 (73.44–78.45)	93.46 (70.12–116.83)	18.36 (-6.68–41.72)	0.14	1,331,963

Abbreviations: AF, atrial fibrillation; SD, standard deviation; ED, emergency department.

[a] Outcomes are all-cause and represent adjusted incremental differences between AF and non-AF groups. Outcomes are all-cause and reported per person-year. Count outcomes (health care utilization; days of sick leave, short-term disability, long-term disability) were modeled with zero-inflated negative binomial regression to address overdispersion and excess zeros. Models adjusted for age, sex, urbanicity, census region, risk factors, and comorbidities. We report adjusted means (95% CIs) by AF status and the adjusted mean difference (AF–non-AF). Wilcoxon nonparametric rank-sum test was used to test the differences in means for continuous variables by AF diagnosis status.

[b] Medical costs were estimated with two-part models and were converted to 2024 USD using the Personal Consumption Expenditures Health Price Index from the U.S. Bureau of Economic Analysis. Because total and component costs were estimated separately with nonlinear links, and laboratory tests were not reported as a standalone category, components may not sum exactly to totals. Outpatient costs and number could not be estimated due to convergence issues.

[c] Dollar values for productivity losses were calculated as: days × 8 hours × the 2021 average hourly wage for private nonfarm employees (U.S. Bureau of Labor Statistics), valuing sick leave at 100% and applying wage-replacement factors of 70% (short-term disability) and 60% (long-term disability). Values were converted to 2024 USD using the Employment Cost Index (Total Compensation for Civilian Workers) from the U.S. Bureau of Labor Statistics.

© 2026 Zhang H et al. *JAMA Network Open*.

eTable 10. Adjusted Medical Costs and Productivity Losses Associated with Atrial Fibrillation Using IPW[a]

Category	Without AF (95% CI)	With AF (95% CI)	Difference [With AF-Without AF] (95% CI)	p-value	N
**Panel A. Medical Costs[b]**					
Total Medical Costs, $	5,758.51 (5,613.13–5,903.89)	24,129.74 (21,218.27–27,041.21)	18,371.22 (15,562.83–21,179.61)	<.001	1,612,398
ED costs, $	519.18 (501.48–536.87)	2,138.34 (1,879.83–2,396.85)	1,619.16 (1,369.46–1,868.87)	<.001	1,612,398
Inpatient costs, $	1,480.69 (1,239.47–1,721.92)	7,142.45 (5,049.79–9,235.09)	5,661.75 (3,717.43–7,606.08)	<.001	1,612,398
Outpatient costs, $	3,468.53 (3,390.53–3,546.53)	14,875.41 (13,344.69–16,406.12)	11,406.87 (9,835.14–12,978.62)	<.001	1,612,398
Pharmacy costs, $	2,365.30 (2,170.57–2,560.03)	4,280.04 (3,793.77–4,766.31)	1,914.74 (1,390.71–2,438.77)	<.001	1,612,398
Number of ED visits	0.21 (0.21–0.22)	0.73 (0.66–0.81)	0.52 (0.45–0.59)	<.001	1,612,398
Number of inpatient admissions	-	-	-	-	-
Number of outpatient visits	9.30 (9.22–9.39)	18.49 (17.61–19.36)	9.19 (8.32–10.05)	<.001	1,612,398
Number of pharmacy prescriptions	-	-	-	-	-
**Panel B. Productivity Loss[c]**					
Num. of sick leave	2.52 (2.25–2.80)	4.00 (2.71–5.28)	1.47 (0.23–2.72)	0.02	139,490
Num. of short-term disability	3.42 (3.24–3.61)	5.99 (4.82–7.15)	2.56 (1.42–3.70)	<.001	1,355,064
Num. of long-term disability	0.52 (0.41–0.64)	0.56 (0.36–0.77)	0.05 (–0.19–0.27)	0.73	1,331,963
Sick leave payment, $	700.97 (625.86–778.84)	1,112.64 (753.81–1,468.69)	408.89 (63.97–756.59)	0.02	139,490
Short-term disability payment, $	665.91 (630.87–702.91)	1,166.32 (938.52–1,392.18)	498.46 (276.49–720.43)	<.001	1,355,064
Long-term disability payment, $	86.78 (68.43–106.81)	93.46 (60.08–128.50)	8.35 (-31.71–45.06)	0.73	1,331,963

Abbreviations: AF, atrial fibrillation; SD, standard deviation; ED, emergency department; IPW, inverse probability weighting.

[a] Outcomes are all-cause and reported per person-year. AF and non-AF groups were balanced using inverse probability weighting. Count outcomes (health care utilization; days of sick leave, short-term disability, long-term disability) were modeled with zero-inflated negative binomial regression to address overdispersion and excess zeros. Models adjusted for age, sex, urbanicity, census region, risk factors, and comorbidities. We report adjusted means (95% CIs) by AF status and the adjusted mean difference (AF–non-AF). Wilcoxon nonparametric rank-sum test was used to test the differences in means for continuous variables by AF diagnosis status.

[b] Medical costs were estimated with two-part models and were converted to 2024 USD using the Personal Consumption Expenditures Health Price Index from the U.S. Bureau of Economic Analysis. Because total and component costs were estimated separately with nonlinear links, and laboratory tests were not reported as a standalone category, components may not sum exactly to totals.

[c] Dollar values for productivity losses were calculated as: days × 8 hours × the 2021 average hourly wage for private nonfarm employees (U.S. Bureau of Labor Statistics), valuing sick leave at 100% and applying wage-replacement factors of 70% (short-term disability) and 60% (long-term disability). Values were converted to 2024 USD using the Employment Cost Index (Total Compensation for Civilian Workers) from the U.S. Bureau of Labor Statistics. Number of inpatient visits and pharmacy prescriptions could not be estimated due to convergence issues.

© 2026 Zhang H et al. *JAMA Network Open*.

eTable 11. Adjusted Medical Costs and Productivity Losses Associated with Atrial Fibrillation Under ATT-Targeted Weights[a]

Category	Without AF (95% CI)	With AF (95% CI)	Difference [With AF-Without AF] (95% CI)	p-value	N
**Panel A. Medical Costs[b]**					
**Total Medical Costs, $**	8,404.00 (8,118.56–8,689.44)	19,558.01 (18,645.47–20,470.55)	11,154.01 (10,420.65–11,887.38)	<.001	1,612,398
ED costs, $	527.16 (805.70–958.30)	1,135.45 (1,589.61–1,888.75)	608.29 (746.88–967.47)	<.001	1,612,398
Inpatient costs, $	1,355.00 (1,329.75–1,380.23)	3,598.91 (3,323.13–3,874.69)	2,243.92 (1,964.56–2,523.28)	<.001	1,612,398
Outpatient costs, $	4,650.18 (4,498.80–4,801.57)	11,524.05 (10,957.29–12,090.82)	6,873.87 (6,389.74–7,358.01)	<.001	1,612,398
Pharmacy costs, $	2,281.62 (2,191.54–2,371.70)	4,121.08 (3,781.37–4,460.79)	1,839.46 (1,554.66–2,124.27)	<.001	1,612,398
Number of ED visits	0.41 (0.40 – 0.42)	0.69 (0.66–0.72)	0.28 (0.25–0.31)	<.001	1,612,398
Number of inpatient admissions	0.23 (0.23–0.24)	0.40 (0.39–0.41)	0.17 (0.16–0.18)	<.001	1,612,398
Number of outpatient visits	24.97 (24.81–25.12)	33.33 (32.89–33.76)	8.36 (7.91–8.81)	<.001	1,612,398
Number of pharmacy prescriptions	17.15 (17.01–17.30)	24.41 (24.01–24.82)	7.26 (6.87–7.66)	<.001	1,612,398
**Panel B. Productivity Loss[c]**					
Num. of sick leave	5.12 (4.43–5.82)	6.00 (4.92–7.09)	0.88 (−0.11–1.86)	0.08	139,490
Num. of short-term disability	9.75 (9.43–10.08)	12.64 (11.85–13.43)	2.89 (2.05–3.73)	<.001	1,355,064
Num. of long-term disability	2.39 (2.10–2.68)	2.78 (2.19–3.38)	0.39 (−0.27–1.06)	0.24	1,331,963
Sick leave payment, $	1,424.18 (1,232.25–1,618.89)	1,668.96 (1,368.55–1,972.15)	244.78 (-30.60–517.38)	0.08	139,490
Short-term disability payment, $	1,898.45 (1,834.78–1,963.06)	2,461.16 (2,307.40–2,613.79)	562.71 (399.10–725.96)	<.001	1,355,064
Long-term disability payment, $	398.88 (350.48–447.28)	463.97 (365.50–564.11)	65.09 (-45.06–176.91)	0.24	1,331,963

Abbreviations: AF, atrial fibrillation; SD, standard deviation; ED, emergency department; ATT, average treatment effect on the treated.

[a] Outcomes are all-cause and reported per person-year. AF and non-AF groups were balanced under ATT-targeted weighting. Count outcomes (health care utilization; days of sick leave, short-term disability, long-term disability) were modeled with zero-inflated negative binomial regression to address overdispersion and excess zeros. Models adjusted for age, sex, urbanicity, census region, risk factors, and comorbidities. We report adjusted means (95% CIs) by AF status and the adjusted mean difference (AF–non-AF). Wilcoxon nonparametric rank-sum test was used to test the differences in means for continuous variables by AF diagnosis status.

[b] Medical costs were estimated with two-part models and were converted to 2024 USD using the Personal Consumption Expenditures Health Price Index from the U.S. Bureau of Economic Analysis. Because total and component costs were estimated separately with nonlinear links, and laboratory tests were not reported as a standalone category, components may not sum exactly to totals.

[c] Dollar values for productivity losses were calculated as: days × 8 hours × the 2021 average hourly wage for private nonfarm employees (U.S. Bureau of Labor Statistics), valuing sick leave at 100% and applying wage-replacement factors of 70% (short-term disability) and 60% (long-term disability). Values were converted to 2024 USD using the Employment Cost Index (Total Compensation for Civilian Workers) from the U.S. Bureau of Labor Statistics. Number of inpatient visits and pharmacy prescriptions could not be estimated due to convergence issues.

© 2026 Zhang H et al. *JAMA Network Open*.

eTable 12. Adjusted Differences in Medical Costs and Productivity Losses Among Individuals with Atrial Fibrillation, Stratified by Sex[a]

Category	Sex	Without AF (95% CI)	With AF (95% CI)	Difference [AF vs. no AF] (95% CI)	p-value (AF vs. no AF)	Difference [Female vs. Male (95% CI)]	p-value (Female vs. Male (95% CI))	N
**Panel A. Medical Costs[b]**								
Total medical cost, $	Female	8,845.40 (8,551.49–9,139.30)	20,776.23 (19,230.11–22,322.35)	11,930.83 (10,486.54–13,375.13)	<.001	997.33 (-549.12–2,543.78)	0.21	623,335
	Male	7,752.20 (7,493.23–8,011.18)	18,685.70 (17,755.61–19,615.78)	10,933.50 (10,149.84–11,717.17)	<.001			989,063
ED cost, $	Female	961.20 (875.65–1,046.75)	2,148.88 (1,886.59–2,411.16)	1,187.68 (956.57–1,418.79)	<.001	422.61 (178.32–666.89)	<.001	623,335
	Male	767.70 (705.98–829.43)	1,532.77 (1,394.72–1,670.82)	765.07 (653.08–877.05)	<.001			989,063
Inpatient cost, $	Female	1,965.28 (1,760.69–2,169.89)	6,756.17 (5,589.62–7,922.73)	4,790.89 (3,684.99–5,896.79)	<.001	1,588.67 (466.23–2,711.12)	0.006	623,335
	Male	2,141.90 (1,954.17–2,329.63)	5,344.12 (4,716.35–5,971.90)	3,202.22 (2,686.33–3,718.11)	<.001			989,063
Outpatient cost, $	Female	5,310.69 (5,137.23–5,484.15)	11,451.69 (10,512.06–12,391.32)	6,141.00 (5,244.53–7,037.47)	<.001	-960.16 (-1,966.23–45.91)	0.06	623,335
	Male	4,184.13 (4,048.34–4,319.94)	11,285.29 (10,670.86–11,899.74)	7,101.16 (6,556.01–7,646.31)	<.001			989,063
Pharmacy prescription cost, $	Female	2,378.62 (2,276.71–2,480.52)	4,309.07 (3,852.66–4,765.46)	1,930.45 (1,497.70–2,363.21)	<.001	93.65 (-395.70–583.00)	0.71	623,335
	Male	2,165.46 (2,083.22–2,247.71)	4,002.26 (3,651.04–4,353.49)	1,836.80 (1,529.36–2,144.23)	<.001			989,063
Numbers of ED visits	Female	0.46 (0.45–0.48)	0.87 (0.80–0.94)	0.40 (0.34–0.47)	<.001	0.14 (0.07–0.22)	<.001	623,335
	Male	0.38 (0.37–0.39)	0.64 (0.61–0.67)	0.26 (0.23–0.29)	<.001			989,063
Numbers of inpatients admissions	Female	0.20 (0.20–0.21)	0.40 (0.37–0.43)	0.20 (0.17–0.23)	<.001	0.04 (0.013–0.075)	0.005	623,335
	Male	0.20 (0.20–0.21)	0.36 (0.35–0.37)	0.15 (0.14–0.17)	<.001			989,063
Numbers of outpatient visits	Female	19.44 (19.26–19.61)	27.90 (26.93–28.87)	8.47 (7.49–9.44)	<.001	1.35 (0.29–2.42)	0.01	623,335
	Male	15.72 (15.59–15.84)	22.83 (22.40–23.25)	7.11 (6.69–7.53)	<.001			989,063
	Female	26.19 (25.99–26.38)	35.57 (34.48–36.67)	9.39 (8.28–10.50)	<.001	1.11 (-0.12–2.34)	0.08	623,335

© 2026 Zhang H et al. *JAMA Network Open*.

Number of pharmacy prescriptions	Male	23.54 (23.40–23.67)	31.81 (31.34–32.28)	8.28 (7.79–8.77)	<.001			989,063
**Panel B. Productivity Loss[c]**								
Number of days of sick absences	Female	5.24 (4.40–6.07)	6.73 (4.50–8.96)	1.50 (-0.70–3.69)	0.18	0.63 (-1.82–3.08)	0.61	623,335
	Male	4.73 (4.13–5.34)	5.60 (4.46–6.74)	0.87 (-0.20–1.93)	0.11			989,063
Number of days of short-term disability	Female	10.52 (9.95–11.09)	14.69 (12.98–16.41)	4.17 (2.52–5.82)	<.001	1.55 (-0.14–3.24)	0.07	513,879
	Male	8.58 (8.29–8.87)	11.20 (10.41–12.00)	2.62 (1.80–3.45)	<.001			841,185
Number of days of long-term disability	Female	3.13 (2.52–3.74)	4.07 (2.71–5.44)	0.94 (-0.33–2.21)	0.15	0.68 (-0.47–1.83)	0.25	530,031
	Male	1.90 (1.64–2.17)	2.17 (1.63–2.71)	0.27 (-0.30–0.83)	0.36			801,932
Sick leave payment, $	Female	1,457.56 (1,223.90–1,688.43)	1,872.01 (1,251.72–2,492.31)	417.24 (-194.72–1,026.41)	0.18	175.24 (-506.25–856.73)	0.61	623,335
	Male	1,315.69 (1,148.80–1,485.37)	1,557.70 (1,240.59–1,874.80)	242.00 (-55.64–536.85)	0.11			989,063
Short-term disability payment, $	Female	2,048.37 (1,937.38–2,159.35)	2,860.32 (2,527.36–3,195.23)	811.94 (490.68–1,133.22)	<.001	301.80 (-27.51–630.77)	0.07	513,879
	Male	1,670.63 (1,614.16–1,727.09)	2,180.77 (2,026.95–2,336.54)	510.15 (350.48–671.76)	<.001			841,185
Long-term disability payment, $	Female	522.38 (420.58–624.46)	679.27 (452.28–908.46)	156.88 (-55.08–368.84)	0.15	113.32 (-78.31–304.92)	0.25	530,031
	Male	317.10 (272.70–361.46)	361.46 (272.04–452.28)	45.06 (-50.07–138.52)	0.36			801,932

Abbreviations: AF, atrial fibrillation; SD, standard deviation; ED, emergency department.

[a] Outcomes are all-cause and reported per person-year. AF and non-AF groups were balanced using propensity-score overlap weighting. Count outcomes (health care utilization; days of sick leave, short-term disability, long-term disability) were modeled with zero-inflated negative binomial regression to address overdispersion and excess zeros. Models adjusted for age, sex, urbanicity, census region, risk factors, and comorbidities. We report adjusted means (95% CIs) by AF status, the adjusted mean difference (AF–non-AF), and adjusted difference between subgroups (95% CIs). Wilcoxon nonparametric rank-sum test was used to test the differences by AF diagnosis status, and Wald test to test the differences between female and male subgroups.

[b] Medical costs were estimated with two-part models and were converted to 2024 USD using the Personal Consumption Expenditures Health Price Index from the U.S. Bureau of Economic Analysis. Because total and component costs were estimated separately with nonlinear links, and laboratory tests were not reported as a standalone category, components may not sum exactly to totals.

[c] Dollar values for productivity losses were calculated as: days × 8 hours × the 2021 average hourly wage for private nonfarm employees (U.S. Bureau of Labor Statistics), valuing sick leave at 100% and applying wage-replacement factors of 70% (short-term disability) and 60% (long-term disability). Values were converted to 2024 USD using the Employment Cost Index (Total Compensation for Civilian Workers) from the U.S. Bureau of Labor Statistics.

© 2026 Zhang H et al. *JAMA Network Open*.

eTable 13. Adjusted Differences in Medical Costs and Productivity Losses Among Individuals with Atrial Fibrillation, Stratified by Rurality [a]

Category	Rurality	Without AF (95% CI)	With AF (95% CI)	Difference [AF vs. no AF] (95% CI)	p-value (AF vs. no AF)	Difference [Urban vs. Rural (95% CI)]	p-value (Urban vs. Rural (95% CI))	N
**Panel A. Medical Costs[b]**								
Total medical cost, $	Urban	8,081.02 (7,810.41–8,351.62)	19,362.11 (18,426.14–20,298.07)	11,281.09 (10,509.59–12,052.60)	<.001	-1,454.84 (-3,592.09–682.42)	0.18	1,489,709
	Rural	9,032.43 (8,651.74–9,413.10)	21,768.35 (19,651.15–23,885.54)	12,735.93 (10,670.29–14,801.56)	<.001			122,689
ED cost, $	Urban	854.84 (781.72–927.96)	1,726.27 (1,575.09–1,877.46)	871.44 (759.11–983.75)	<.001	-210.44 (-614.42–193.55)	0.31	1,489,709
	Rural	926.44 (838.42–1,014.45)	2,008.31 (1,603.26–2,413.37)	1,081.87 (683.90–1,479.85)	<.001			122,689
Inpatient cost, $	Urban	2,137.67 (1,942.20–2,333.15)	5,664.61 (4,998.80–6,330.42)	3,526.93 (2,987.11–4,066.76)	<.001	-1,265.80 (-2,748.00–216.41)	0.09	1,489,709
	Rural	2,348.54 (2,057.91–2,639.18)	7,141.28 (5,627.39–8,655.17)	4,792.74 (3,320.67–6,264.80)	<.001			122,689
Outpatient cost, $	Urban	4,487.80 (4,344.42–4,631.18)	11,499.73 (10,907.13–12,092.33)	7,011.93 (6,495.76–7,528.10)	<.001	-647.71 (-2,150.62–855.19)	0.40	1,486,336
	Rural	5,200.51 (4,976.88–5,424.13)	12,860.15 (11,395.18–14,325.13)	7,659.64 (6,216.54–9,102.75)	<.001			122,615
Pharmacy prescription cost, $	Urban	2,245.62 (2,163.66–2,327.57)	4,174.04 (3,835.27–4,512.80)	1,928.43 (1,632.40–2,224.45)	<.001	556.47 (25.43–1,087.53)	0.04	1,489,709
	Rural	2,204.42 (2,058.05–2,350.79)	3,576.36 (3,118.98–4,033.75)	1,371.95 (909.51–1,834.38)	<.001			122,689
Numbers of ED visits	Urban	0.39 (0.38–0.40)	0.68 (0.65–0.71)	0.28 (0.25–0.31)	<.001	-0.05 (-0.14–0.039)	0.26	1,489,709
	Rural	0.42 (0.40–0.44)	0.76 (0.67–0.84)	0.34 (0.25–0.42)	<.001			122,689
Numbers of inpatients admissions	Urban	0.21 (0.20–0.21)	0.37 (0.35–0.38)	0.16 (0.15–0.17)	<.001	-0.035 (-0.078–0.008)	0.11	1,489,709
	Rural	0.19 (0.18–0.20)	0.38 (0.35–0.42)	0.20 (0.15–0.24)	<.001			122,689
Numbers of outpatient visits	Urban	16.63 (16.51–16.76)	24.09 (23.67–24.51)	7.46 (7.04–7.87)	<.001	0.61 (-0.64–1.86)	0.34	1,489,709
	Rural	15.13 (14.88–15.38)	21.98 (20.82–23.13)	6.85 (5.67–8.03)	<.001			122,689
	Urban	23.85 (23.72–23.98)	32.28 (31.83–32.73)	8.43 (7.96–8.89)	<.001	-0.80 (-2.42–0.82)	0.34	1,489,709

© 2026 Zhang H et al. *JAMA Network Open*.

Number of pharmacy prescriptions	Rural	26.32 (25.98–26.66)	35.54 (34.04–37.05)	9.22 (7.68–10.76)	<.001			122,689
**Panel B. Productivity Loss[c]**								
Number of days of sick absences	Urban	4.66 (4.03–5.29)	5.56 (4.51–6.61)	0.90 (-0.04–1.85)	0.06	-0.84 (-5.63–3.95)	0.73	127,928
	Rural	6.18 (5.22–7.13)	7.92 (3.34–12.49)	1.74 (-2.97–6.44)	0.47			11,562
Number of days of short-term disability	Urban	9.02 (8.75–9.29)	11.83 (11.05–12.62)	2.81 (2.00–3.63)	<.001	-1.10 (-3.39–1.19)	0.35	1,251,290
	Rural	8.70 (7.89–9.52)	12.61 (10.26–14.97)	3.91 (1.67–6.16)	<.001			103,774
Number of days of long-term disability	Urban	2.16 (1.92–2.40)	2.57 (1.99–3.15)	0.41 (-0.21–1.03)	0.19	0.061 (-1.15–1.27)	0.92	1,231,554
	Rural	2.22 (1.33–3.11)	2.57 (1.05–4.09)	0.35 (-0.90–1.60)	0.58			100,409
Sick leave payment, $	Urban	1,296.22 (1,120.99–1,471.47)	1,546.57 (1,254.50–1,838.64)	250.34 (-11.13–514.60)	0.06	-233.66 (-1,566.03–1,098.74)	0.73	127,928
	Rural	1,719.03 (1,451.99–1,983.27)	2,203.14 (929.05–3,474.22)	484.00 (-826.13–1,791.35)	0.47			11,562
Short-term disability payment, $	Urban	1,756.30 (1,703.73–1,808.87)	2,303.44 (2,151.57–2,457.26)	547.14 (389.42–706.80)	<.001	-214.19 (-660.07–231.71)	0.35	1,251,290
	Rural	1,693.99 (1,536.27–1,853.66)	2,455.32 (1,997.75–2,914.84)	761.33 (325.16–1,199.42)	<.001			103,774
Long-term disability payment,$	Urban	360.49 (320.44–400.55)	428.92 (332.13–525.72)	68.43 (-35.04–171.91)	0.19	10.18 (-191.93–211.96)	0.92	1,231,554
	Rural	370.51 (221.97–519.05)	428.92 (175.24–682.61)	58.41 (-150.21–267.03)	0.58			100,409

Abbreviations: AF, atrial fibrillation; SD, standard deviation; ED, emergency department.

[a] Outcomes are all-cause and reported per person-year. AF and non-AF groups were balanced using propensity-score overlap weighting. Count outcomes (health care utilization; days of sick leave, short-term disability, long-term disability) were modeled with zero-inflated negative binomial regression to address overdispersion and excess zeros. Models adjusted for age, sex, urbanicity, census region, risk factors, and comorbidities. We report adjusted means (95% CIs) by AF status, the adjusted mean difference (AF–non-AF), and adjusted difference between subgroups (95% CIs). Wilcoxon nonparametric rank-sum test was used to test the differences by AF diagnosis status, and Wald test to test the differences between urban and rural subgroups.

[b] Medical costs were estimated with two-part models and were converted to 2024 USD using the Personal Consumption Expenditures Health Price Index from the U.S. Bureau of Economic Analysis. Because total and component costs were estimated separately with nonlinear links, and laboratory tests were not reported as a standalone category, components may not sum exactly to totals.

[c] Dollar values for productivity losses were calculated as: days × 8 hours × the 2021 average hourly wage for private nonfarm employees (U.S. Bureau of Labor Statistics), valuing sick leave at 100% and applying wage-replacement factors of 70% (short-term disability) and 60% (long-term disability). Values were converted to 2024 USD using the Employment Cost Index (Total Compensation for Civilian Workers) from the U.S. Bureau of Labor Statistics.

© 2026 Zhang H et al. *JAMA Network Open*.
