# Supplementary material for: Medical Costs and Productivity Losses of Atrial Fibrillation Among US Privately Insured Employees
Source: JAMA Netw Open. 2026 Feb 12;9(2):e2559227. doi: 10.1001/jamanetworkopen.2025.59227 (PMC12902891; doi:10.1001/jamanetworkopen.2025.59227)
Supplement: Supplement 2. — Data Sharing Statement [file jamanetwopen-e2559227-s002.pdf]

Data Sharing Statement

Zhang. Medical Costs, Productivity Losses, and Atrial Fibrillation Among US Privately Insured Employees. *JAMA Netw Open*. Published February 12, 2026. doi:10.1001/jamanetworkopen.2025.59227

Data
Data available: Yes
Data types: Other (please specify)
Additional Information: analytic code and accompanying documentation
How to access data: The data underlying this study are deidentified administrative claims and employer absence records licensed from the Merative MarketScan Commercial and Health and Productivity Management databases and are not publicly available under the terms of the data use agreement. The authors are not permitted to share these data. Qualified investigators may request access directly from the data provider (subject to institutional approvals, licensing fees, and a data use agreement). The analytic code and accompanying documentation will be made available on the project's GitHub repository upon publication and can be obtained from the corresponding author prior to publication.
When available: With publication

Supporting Documents
Document types: Statistical/analytic code
How to access documents: The analytic code and documentation will be posted to the project's GitHub repository with publication (URL to be provided then). Prior to publication, materials can be requested from the corresponding author's email.
When available: With publication

Additional Information
Who can access the data: Analytic code and documentation: available to anyone (public GitHub); prior to publication, available on request from the corresponding author. Underlying licensed datasets: available only from the data provider to qualified investigators whose requests are approved and who enter into a data use agreement.
Types of analyses: Analytic code and documentation: for replication and methodological transparency, and for reasonable secondary analyses or extensions of the study methods. Underlying licensed datasets: any analyses permitted by the data provider's data use agreement and institutional approvals.
Mechanisms of data availability: Analytic code and documentation: Publicly available without investigator support via the project's GitHub repository. Underlying licensed datasets: From the data provider, not the authors, with a signed data use agreement.
